# How the brain memorizes the world from others’ perspectives: investigating allocentric encoding of object features during perspective taking

**DOI:** 10.1186/s40359-025-03022-2

**Published:** 2025-07-01

**Authors:** Nanbo Wang, Shuo Huang, Junyang Cai, Ruiyi Huang, Haiyan Geng

**Affiliations:** 1 Department of Psychology, School of Health, Fujian Medical University, Fuzhou, 350122 China; 2 School of Psychological and Cognitive Sciences, Beijing Key Laboratory of Behavior and Mental Health, Peking University, Beijing, 100871 China

**Keywords:** Working memory, Perspective taking, Object-based encoding, Feature-based encoding, Other-perspective processing

## Abstract

**Background:**

Individuals are frequently encouraged to consider others’ perspectives to enhance social interactions. To achieve this objective, understanding others’ visual world is significantly beneficial. However, as a fundamental aspect of human cognition, to what extent can individuals effectively memorize object information when adopting another’s viewpoint? Can all features about the object be exclusively memorized in an allocentric manner or only some of them? The present study aims to investigate this issue.

**Methods:**

We designed a novel paradigm by integrating a change-detection paradigm with a perspective taking task to investigate these questions. A total of 226 undergraduates were recruited in four behavioral experiments. Participants were instructed to memorize the target dimension features of a memory array from an avatar’s perspective while ignoring irrelevant dimension features. We assessed the impact of irrelevant feature changes on individuals’ identification of memorized target features to determine whether irrelevant features are automatically memorized in an allocentric manner.

**Results:**

We found that individuals engaged in other-perspective processing can adopt either object-based encoding, where irrelevant features are automatically encoded into memory in an allocentric manner along with the target feature, or feature-based encoding, where irrelevant features will not be memorized without controlled intention. The selection of encoding mechanism depends on the discriminability of task-irrelevant dimensions. Object-based encoding is employed when the irrelevant dimension exhibits high discriminability while feature-based encoding is utilized when it demonstrates low discriminability. Moreover, these mechanisms are specific to embodied processing, and alternative strategies like object-rotation will lead to different encoding rules.

**Conclusions:**

Overall, this study revealed that individuals follow a “coarse-to-fine” principle of memorizing object features when adopting others’ perspectives and suggests the existence of allocentric memorization of irrelevant information during perspective taking.

**Supplementary Information:**

The online version contains supplementary material available at 10.1186/s40359-025-03022-2.

## Introduction

Interpersonal communication constitutes the underpinnings of normal functioning within human society, relying on individuals’ cognitive capacity to comprehend others’ thoughts and intentions [14, 25, 34]. This ability, referred to as perspective taking, is critical not only for everyday interactions but also for navigating complex social scenarios such as negotiating [15, 49], training [22], education [35], and even medical treatment [2]. To gain a comprehensive understanding of how we infer others’ mental states, it is crucial to investigate the underlying mechanisms involved in perspective taking. A prominent theoretical framework, the stage model, postulates that information processing involves a series of stages that transform stimulus information into observable responses [46]. To react to a stimulus, we must first perceptually encode it, match its perceptual representation with stored knowledge in memory, and then select and execute an appropriate behavioral response [38]. Nevertheless, despite considerable research into self-perspective perceptual processing, our understanding of how we process, represent, and memorize visual information from others’ perspectives remains largely unknown. How do we memorize the world when we switch to an allocentric perspective to facilitate social interaction? This question is essential for comprehending others’ mental landscapes and relates to the fundamentals of how the human brain functions across different perspectives to represent the world. Given that encoding object features and storing them in memory are crucial aspects of human cognition [31, 51], this study aims to investigate the above questions by examining how information is encoded and memorized when adopting others’ perspectives.

In self-perspective processing, individuals may employ two distinct mechanisms for encoding object features based on different situations. On the one hand, visual processing involves object-based encoding (OBE), wherein task-irrelevant features of an object are automatically extracted into working memory whenever task-related features belonging to the same object are processed, irrespective of the observer’s intention [20, 23, 45]. This encoding mechanism reflects the hierarchical organization prevalent in visual working memory, where individual features are integrated into chunks to enhance memory efficiency [13, 36]. Correspondingly, in a change-detection task, the alteration of task-irrelevant features can significantly disrupt individuals’ responses to the target feature, resulting in the irrelevant-change distracting effect [59, 61]. The OBE is usually noticeable when the task-irrelevant feature possesses “coarse” information with high discriminability [23], such as basic color and location, and it has consistently been observed across various exposure times [45] and under both low and high memory load conditions [59].

In contrast, memory encoding can also be feature-based, whereby only the selected feature enters working memory while the other task-irrelevant features are disregarded [1, 47, 55, 56]. Consequently, changes in task-irrelevant features do not hinder individuals’ responses to the target feature. This form of encoding (known as feature-based encoding, FBE) is typically observed when the task-irrelevant features hold “fine” information with low discriminability, such as complex color and orientation [19]. Neurophysiological evidence indicates that cortical neurons prioritize the representation of task-relevant features over task-irrelevant ones during memory retention intervals [37, 42]. This prioritization is particularly evident when nonspatial features are irrelevant [41] or when memory load is high [58].

Therefore, individuals employ these two encoding mechanisms according to the “coarse-to-fine” rule, meaning that these mechanisms are not entirely antagonistic. Specifically, “coarse” irrelevant information with high discriminability, such as simple color and location, can be automatically encoded into working memory through OBE. Conversely, “fine” irrelevant information with low discriminability, such as complex color and orientation, cannot be processed automatically and typically results in FBE. The brain selectively utilizes these two mechanisms, thereby facilitating object identification while minimizing cognitive resources [18, 23]. Given that the encoding of objects is fundamental to our recognition of the world, it is crucial to comprehend how we encode and memorize object features from another’s perspective in order to gain insights into how we understand others’ perceptual world. However, to our knowledge, there exists a notable gap in research exploring this specific area. Therefore, the primary objective of this study is to investigate the underlying mechanisms involved in encoding visual information from others’ viewpoints.

We propose that there exist two potential mechanisms for encoding features when adopting others’ perspectives. First, the principles of visual encoding from self-perspective may also apply in other-perspective processing. Several studies suggest that individuals adopt others’ visual-spatial perspectives (visual-spatial perspective taking, VSPT) through an embodied approach [4, 10, 32, 53]. Specifically, individuals mentally transform themselves to the spatial location of the person whose visual perspective is being adopted (known as mental-body transformation) and imagine perceiving the visual world from that viewpoint as if they are physically being in that location [27, 48]. These processes occur within the imagination and depend on the mental imagery of the visual scene [7, 33, 52]. The support for this embodied approach primarily comes from the impact of one’s physical form and related elements, such as bodily posture and handedness, on the speed of VSPT tasks. For instance, participants responded faster in a VSPT task when their body rotations aligned with the required mental-body transformation, rather than being in opposite directions [26, 27]. Additionally, participants demonstrated faster target judgment when the avatar grasped the target with its hand on the ipsilateral side as their dominant hands, compared to when it grasped with the hand on the contralateral side [21]. Consequently, we hypothesize that if individuals process others’ visual worlds using an embodied approach, they are likely to encode perceptual information from others’ perspectives in a manner akin to self-perspectives, due to their immersive processing in the “imagined” others’ locations (the locations of the virtual self).

Importantly, this hypothesis aligns with the self-other shared mechanism proposed by some researchers [28]. Specifically, during self- and other- referential tasks, the processing of stimuli related to oneself and other individuals (e.g., perception, emotion, and movement) may activate overlapping neural circuits [8, 9]. Through these circuits, individuals can comprehend others’ behaviors by associating them with their own sensory-motor experiences that arise when engaging in similar behaviors. For instance, individuals utilize shared neuronal populations to process their own and others’ perihand space [3], while also employing the neural basis of self-body representation for forecasting others’ emotions and perceptions [24]. Consistent with this hypothesis, evidence suggests that the neural mechanisms underlying visual processing from others’ perspectives exhibit overlap with those involved in self-perspective. For instance, Yuan et al. [60] demonstrated that adopting another individual’s visual perspective to perceive motion stimuli elicited similar motion adaptation effects as those observed under one’s own perspective, thereby implying the engagement of a shared neuronal population in processing moving stimuli from both perspectives.

The embodied VSPT approach, along with the shared mechanism hypothesis, leads us to infer that the mechanisms underlying memory encoding from others’ perspectives and self-perspective may exhibit similarities. Specifically, the “coarse-to-fine” rule observed in one’s self-perspective processing may also be applicable to allocentric encoding: when processing from others’ viewpoints, the brain employs either object-based encoding or feature-based encoding based on the discriminability of the task-irrelevant features. Highly discriminable features are automatically extracted into visual working memory in an allocentric manner through OBE, while low-discriminability features cannot be processed automatically and result in FBE.

However, contrary to the notion of shared mechanisms, some researchers have uncovered distinctions between other- and self-perspective processing [6]. For example, individuals can spontaneously adopt another person’s viewpoint to perceive target objects even when those targets are not visible to that person [29, 54]. Additionally, participants did not experience a perceptual difference in length between two identical-sized lines viewed from different distances under others’ viewpoints [39]. These findings highlight the unique characteristics of other-perspective processing and challenge the idea that similar mechanisms are employed for processing across different viewpoints.

Correspondingly, alternative strategies for implementing other-perspective processing are proposed. For instance, the object-rotation strategy entails mentally rotating the target stimuli from others’ viewpoints to one’s own and subsequently responding [40, 48]. Additionally, the perspective-calculation strategy employs spatial cues to establish correspondence between two perspectives (e.g., “if the other person is sitting opposite me, then my right is their left”). Given the substantial cognitive demands of both rotation and calculation approaches, it is plausible to hypothesize that the limited capacity of working memory [31, 51] selectively processes target features and excludes task-irrelevant ones to facilitate efficient processing [50]. Ultimately, irrespective of discriminability, only task-relevant features would be encoded, manifesting as a feature-based encoding.

Furthermore, even with the embodied VSPT approach, the brain may adopt a distinct encoding mechanism when perceiving from others’ perspectives compared to our own perspective. Considering the cognitive demands associated with mental-body transformation, it is plausible that limitations in cognitive resources may arise, leading to the adoption of feature-based encoding irrespective of irrelevant feature discriminability. Collectively, these possibilities suggest that different mechanisms may exist for encoding features from alternative perspectives as compared to our own.

In this study, we aim to investigate how individuals encode and memorize object information from another person’s perspective during VSPT. To achieve this objective, we employed a modified change-detection task where participants adopted an avatar’s viewpoint to memorize a specific feature dimension (target dimension) of the presented memory array. Then, they judged whether a specific feature in the target dimension (target feature) of the subsequently presented probe stimulus appeared in the memorized array. Notably, we manipulated changes in the feature of the irrelevant dimension of the probe stimulus (irrelevant feature). We propose that impaired behavioral responses resulting from irrelevant feature changes indicate the encoding of all feature dimensions in the memory array (OBE), while nonimpaired performance supports the encoding of only task-relevant dimension (FBE). In Experiment 1, our findings provide support for the self-other overlap hypothesis and indicate that when adopting others’ perspectives, an OBE mechanism is employed when the task-irrelevant feature has high discriminability, while a FBE approach is utilized when the irrelevant feature has low discriminability. Additionally, Experiments 2 and 3 ruled out alternative explanations and confirm that the employment of the two encoding manners conforms to the “coarse-to-fine” rule. Finally, Experiment 4 corroborates participants’ adherence to the instructed embodied approach and reveals distinct encoding mechanisms employed during mental-body transformation and an alternative (object-rotation) strategy.

### Experiment 1

To examine the encoding mechanisms employed when processing from others’ perspectives, we conducted an experiment on change-detection from the viewpoint of an avatar. Participants adopted an avatar’s perspective to memorize features in the target dimension of a memory array while ignoring those in the irrelevant dimension. To ensure that participants adopted the avatar’s perspective rather than processing stimuli from their own perspectives, we utilized spatial features with distinct appearances from different viewpoints. Specifically, the memory array comprised arrows in various locations and orientations, thereby incorporating two spatial dimensions. One dimension was designated as the target dimension, while the other served as the irrelevant dimension or this relationship was reversed. Given that orientation is a less discriminable dimension and location is a highly discriminable one, investigating the perceptual processing of these features from an allocentric perspective can shed light on whether it conforms to the “coarse-to-fine” rule. Our hypothesis was that if individuals employ similar mechanism to memorize feature information from both others’ perspectives and their own, they will exhibit OBE when the irrelevant dimension was location and FBE when it was orientation, in accordance with the “coarse-to-fine” rule. Alternatively, if different mechanisms are used for each perspective, a consistent utilization of FBE would be predicted regardless of the irrelevant dimension.

## Methods

### Participants

The sample size of Experiment 1 was determined using G*Power 3 [11] via a priori power analysis. Because no prior research regarding perceptual encoding from others’ perspectives was available, we conservatively estimated a medium effect size (η_p_^2^ = 0.06). With this effect size, a power of 0.80, and an alpha level of 0.05, the power analysis conducted with a 2 × 2 repeated measure ANOVA yielded an estimated sample size of 34. Ultimately, a total of 42 undergraduates were recruited for Experiment 1 (19 males, 23 females, aged 19–21 years, *M* = 19.500, *SD* = 0.594). Each participant received a compensation of 40 RMB for their contribution.

All experiments in this paper were approved by the Ethics Committee at the first author’s university (reference no. 2022–147). Participants were recruited from the same institution. They provided written informed consent prior to participation, and all had normal or corrected-to-normal vision.

### Apparatus and stimuli

The stimuli were presented on a 19-inch CRT monitor (1920 × 1080 pixels, 60 Hz refresh rate) against a gray background (RGB: 150, 150, 150). Participants were seated 60 cm away from the screen. This setup was used consistently throughout the experiments mentioned in the article.

The memory array consisted of three arrows in various orientations and locations, with the features randomly selected from six distinct orientation values (0°, 45°, 135°, 180°, 225°, and 315° relative to the vertical upward direction) and eight locations within a 3 × 3 matrix (excluding the center location), respectively. The arrows were generated by randomly combining feature values from the two dimensions. The avatar was positioned on either the left or right side of the screen while facing the center of the matrix.

### Design and procedure

Experiment 1 adopted a 2 (Target Dimension: Location vs. Orientation) × 2 (Irrelevant Feature: No change vs. Change) within-participant design. Each target dimension was conducted in separate sessions, namely the *location* session and the *orientation* session. Each session comprised four blocks, where the avatar appeared on both the left and right sides of the screen for two blocks each. Each block consisted of 40 trials, resulting in a total of 320 formal trials. To ensure familiarity with the procedure, participants completed at least 12 practice trials before each session.

**Fig. 1:**
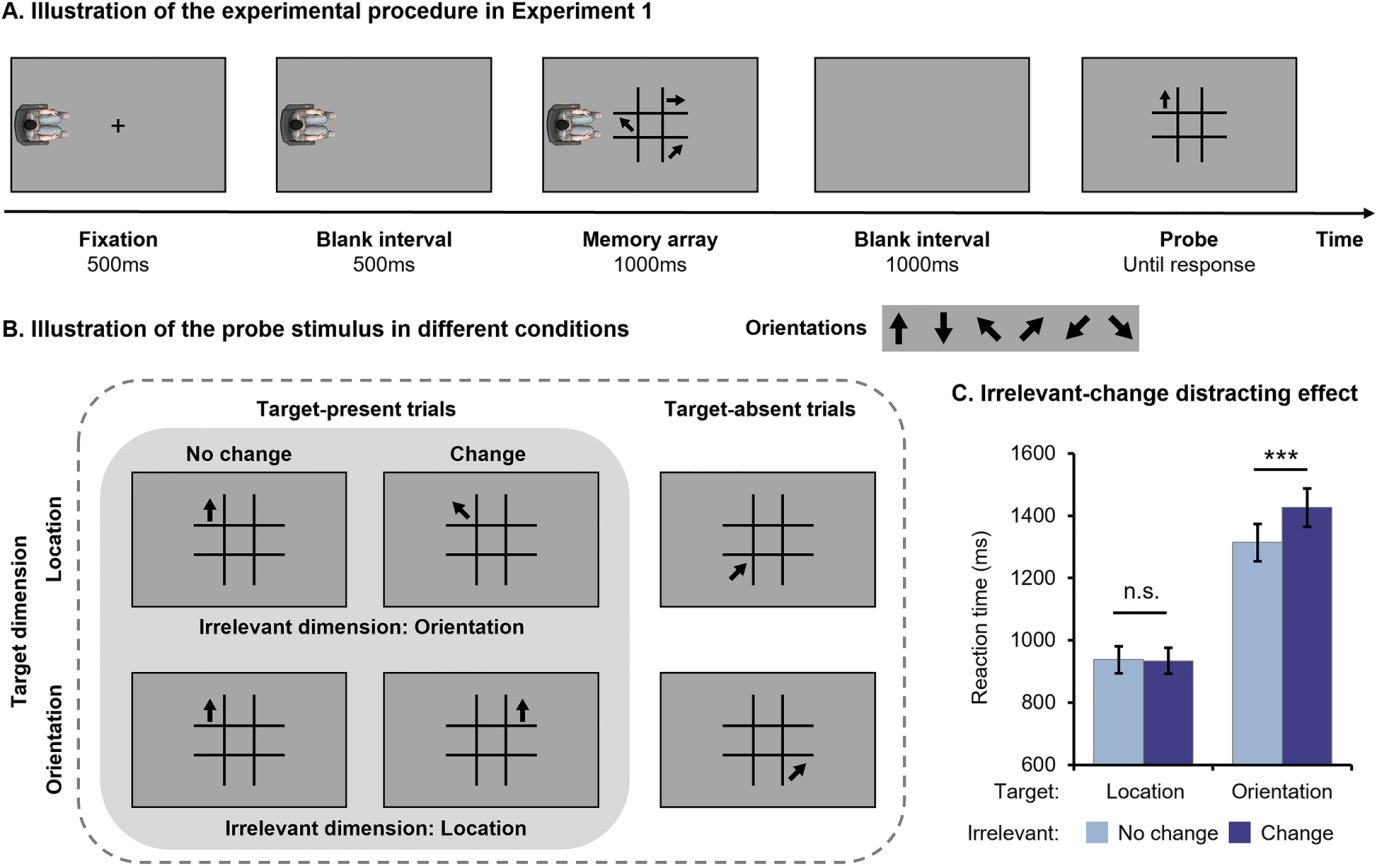
Illustration of experimental procedures (**A**), design (**B**), and results (**C**) for Experiment 1. Error bars represent one standard error. Significant pairwise comparisons are denoted by asterisks (*p < 0.05, **p < 0.01, ***p < 0.001), and the notation “n.s.” indicates lack of statistical significance. These notations apply to all figures included in this paper

Each trial started with the appearance of an avatar and a 500 ms fixation cross (a black cross positioned at the center of the screen, 0.5° × 0.5°). The fixation was then removed for 500 ms. Subsequently, a memory array consisting of three arrows arranged in a 3 × 3 matrix was displayed for 1000 ms (see Fig. 1A). Participants were instructed to imagine themselves being in the avatar’s position and attentively observe and memorize target dimension features of the memory array from the avatar’s perspective. In the location condition, participants were instructed to memorize the locations of the arrows (target dimension) while ignoring their orientations (irrelevant dimension). Conversely, in the orientation condition, participants were required to memorize the arrow orientations and ignore their locations. Then, both the avatar and memory array disappeared. Following a 1000 ms blank interval, a probe stimulus was presented to assess the memory retention of the array. Participants had to determine whether the target feature of the probe stimulus had been previously displayed in the memory array from the avatar’s viewpoint. The irrelevant change or no change condition was determined by comparing the irrelevant feature of the probe stimulus viewed from participants’ self-perspectives (probe stage) with that of the memory array viewed from the avatar’s perspective (encoding stage) in each trial. The participants were instructed to respond as quickly and accurately as possible.

In half of the trials, the probe stimulus contained one of the target features observed from the avatar’s perspective in the preceding memory array. For 50% of these “target-present trials”, we created the probe stimulus by randomly selecting one arrow from the previous memory array and rotating the visual scene, including both the arrow and the matrix, from the avatar’s perspective to the participant’s self-perspective. This manipulation maintained the arrow’s target and irrelevant features unchanged when observed from the avatar’s perspective during the encoding stage and from participants’ self-perspectives during the probe stage (see Fig. 1B). The probe arrow for the remaining 50% of “target-present trials” was generated by blending a target feature with an irrelevant feature from two randomly selected arrows in the prior memory array. Subsequently, the visual scene (including the created arrow and the matrix) underwent rotation as previously described to construct the probe stimulus, thereby ensuring constancy of the target feature while eliciting variations in the irrelevant feature.

The other half of the trials, referred to as “target-absent trials”, did not include the target features from the previous memory array (seen from the avatar’s perspective) in the probe stimulus. Instead, a novel location or orientation not previously observed from the avatar’s perspective was employed as the target feature. In 50% of these trials, the irrelevant feature was randomly selected from the three existing arrows in the memory array viewed by the avatar. For the remaining 50% of trials, the irrelevant feature was a novel feature not presented in the memory array. Given our interest in investigating whether changes in the irrelevant feature affect individuals’ responses in identifying the target feature, we will analyze data from target-present trials to discern the employed encoding mechanism. Additionally, data from target-absent trials will be utilized to uncover participants’ egocentric errors (i.e., mistaking visual features seen from their own perspectives as those seen from the avatar’s perspective) during allocentric processing.

Finally, we incorporated a *both* session as filler trials, wherein participants were asked to memorize both the location and orientation of the arrows. Then, they had to determine if the probe stimulus’s location and orientation matched any of the three arrows in the preceding memory array from the avatar’s perspective. This session was included to prevent participants from suspecting the purpose of the experiment. Note that participants believed the task was a memory test, and assessing memory solely based on one dimension is counterintuitive when compared to employing two dimensions that offer greater evaluation potential.

During this session, half of the probe stimuli consisted of a previously presented arrow in the memory array from the avatar’s perspective, while the other half comprised a novel arrow created by combining two features (a target and an irrelevant feature) extracted from two randomly selected arrows in the array, also from the avatar’s viewpoint. This session was also divided into four 40-trial blocks, with the avatar appearing on the left and right sides of the screen between blocks. The order of the three sessions (*location*, *orientation*, and *both*) and the avatar’s location in the twelve blocks were counterbalanced across participants. Data from filler trials will not be included in the analysis.

## Results

Trials with reaction times exceeding two standard deviations from the mean of their respective conditions were excluded from data analysis. The analysis of the irrelevant-change distracting effect solely focused on data obtained from accurately responded target-present trials. This approach ensures that any differences observed between the No change and Change conditions are exclusively attributed to changes in irrelevant features rather than other confounding factors. This pre-analysis procedure was consistently implemented across all experiments without any further mention.

Participants’ accuracy rates (ACCs) in target-present and target-absent trials were 87.991% (*SD* = 6.752%) and 89.911% (*SD* = 5.703%), respectively. Overall, participants achieved an accuracy rate of 88.951% (*SD* = 5.471%). Considering previous research suggesting that the distracting effect primarily manifests in participants’ RTs rather than their ACCs [19, 45], this paper focuses on reporting RT results. The ACC analyses, which yielded consistent results overall, along with the analyses of participants’ egocentric errors during the task, are included in the Supplementary Material. All *p* values reported in this article underwent Bonferroni correction for multiple comparisons.

We conducted a 2 × 2 repeated-measure ANOVA with Target Dimension (Location vs. Orientation) and Irrelevant Feature (No change vs. Change) as within-participant factors on the RTs of target-present trials. The main effects of Target Dimension (*F* (1, 41) = 116.190, *p* < 0.001, η_p_^2^ = 0.739) and Irrelevant Feature (*F* (1, 41) = 8.197, *p* = 0.007, η_p_^2^ = 0.167), as well as their interaction (*F* (1, 41) = 25.635, *p* < 0.001, η_p_^2^ = 0.385), were all significant. Post-hoc pairwise comparisons revealed that changes in the irrelevant feature significantly slowed participants’ response speed when recognizing the orientation of the probe arrow (*p* < 0.001), but not when recognizing its location (*p* = 0.823, see Fig. 1C and Table 1). On the other hand, participants responded significantly faster when recognizing the location rather than the orientation of the probe arrow, irrespective of any changes to irrelevant features (*ps* < 0.001).

**Table 1:** Reaction times in Experiment 1–4 [*M*(*SE*), in ms]

Experiment	Target Dimension	Irrelevant Feature	
		No change	Change
1	Location	937.8 (43.5)	934.0 (41.5)
	Orientation	1313.7 (59.6)	1426.2 (61.7)
2	Location	906.3 (46.6)	956.2 (49.7)
	Orientation	1247.2 (72.9)	1349.4 (80.6)
3	Orientation	1335.5 (73.5)	1316.5 (68.6)
4		Mental-transformation condition	
	Location	1130.6 (40.4)	1174.6 (45.1)
	Orientation	1366.3 (56.1)	1542.7 (80.6)
		Object-rotation condition	
	Location	1102.5 (62.6)	1139.8 (61.0)
	Orientation	1562.0 (105.3)	1545.5 (129.9)

## Discussion

By manipulating task-irrelevant features in a change-detection task, we found that individuals can employ either object-based or feature-based encoding depending on the discriminability of the irrelevant dimension when adopting another person’s perspective. Specifically, changes in the irrelevant feature significantly impeded participants’ performance in recognizing the target when orientation was treated as the target dimension and location as the irrelevant dimension. This suggests that participants automatically processed the task-irrelevant location dimension from others’ perspectives, and encoded location features into working memory, aligning with the mechanism of OBE. In contrast, when location was the target dimension and orientation was the irrelevant dimension, individuals responded at a comparable speed regardless of orientation changes. This implies that the task-irrelevant orientation dimension was not processed during the memorization of arrow locations from the avatar’s perspective, aligning with the mechanism of FBE. The ACC results further support these findings and indicate that the faster RTs observed in the irrelevant no change condition, compared to the change condition, did not come at the expense of decreased accuracy in the former condition (see Fig. S1). Therefore, these findings suggest a selective employment of two encoding manners in other-perspective visual processing. Considering that orientation is a fine-grained dimension and location is a coarse one, this pattern of utilization is consistent with the “coarse-to-fine” rule.

However, an alternative explanation for our findings should be considered: while the irrelevant dimension varied between the two conditions (orientation vs. location), the target dimensions changed simultaneously (location vs. orientation). Therefore, it is plausible that participants’ encoding was influenced by the attributes of the target dimension rather than those of the irrelevant one. Experiment 2 was designed to investigate this explanation.

### Experiment 2

In Experiment 2, we aimed to test whether the encoding mechanism employed by individuals when adopting others’ perspectives depends on the attributes of the irrelevant dimension rather than those of the target dimension. We utilized a similar paradigm as in Experiment 1 but adopted a modified binding method that incorporated location, orientation, and color. While location and orientation were treated respectively as target dimensions in two distinct conditions, color served as the irrelevant dimension in both conditions. If the attributes of the irrelevant dimension dictate individuals’ encoding mechanism under others’ perspectives, comparable outcomes should be observed under both conditions due to the presence of a consistent irrelevant dimension. Nevertheless, if the attributes of the target dimension also influence perceptual encoding, divergent results are expected between the two conditions due to variations in target dimensions.

## Methods

### Participants

**Fig. 2:**
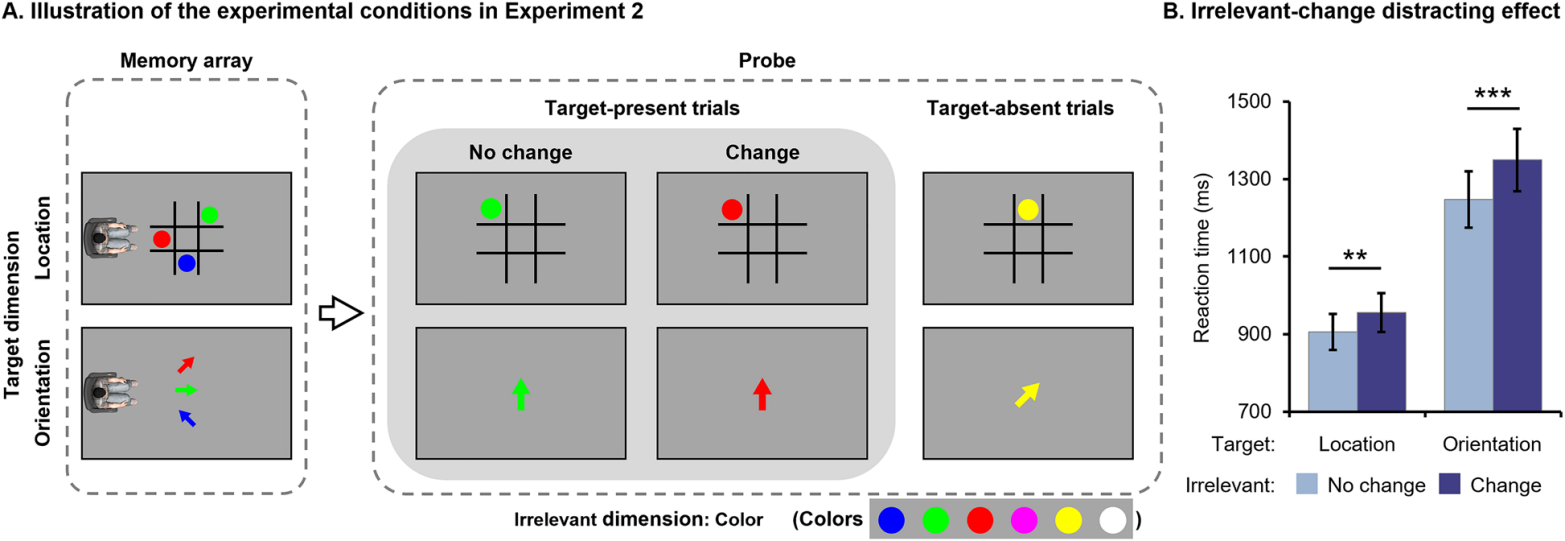
Illustration of experimental conditions (**A**) and results (**B**) for Experiment 2

We conducted a power analysis with G*Power 3 to determine the sample size of Experiment 2. To avoid potential interference between location and orientation, we randomly allocated participants into the location (location-color binding) or the orientation (orientation-color binding) condition (see Fig. 2A). As a between-participant design may cause some additional error variance [5], we conservatively estimated a medium-to-small effect size (η_p_^2^ = 0.03). With this effect size, a power of 0.80 and an alpha level of 0.05, the power analysis conducted with a 2 × 2 mixed measure ANOVA resulted in a minimum sample size of 66.

A total of 78 undergraduates participated in Experiment 2 and were each provided with a compensation of 30 RMB for their participation. Data from two participants were excluded: one for outlier RTs exceeding three standard deviations from the group mean, and the other for outlier accuracy below three standard deviations from the group mean. Data from the remaining 76 participants (35 males and 41 females, aged 18–25 years, *M* = 20.329, *SD* = 1.464) were included for subsequent analyses.

### Stimuli

The memory array consisted of three colored circles in different locations of the matrix (the location condition) or three colored arrows with different orientations (the orientation condition). The colors of these elements were randomly chosen from a set of six distinct color values: blue (0, 0, 255), green (0, 255, 0), red (255, 0, 0), magenta (255, 0, 255), yellow (255, 255, 0), and white (255, 255, 255), provided in RGB format (see Fig. 2A). The circles’ locations and the arrows’ orientations were constructed using the same methodology as in Experiment 1.

### Design and procedure

Experiment 2 employed a mixed design of 2 (Target Dimension: Location vs. Orientation) × 2 (Irrelevant Feature: No change vs. Change), with Target Dimension as the between-participant variable and Irrelevant Feature as the within-participant variable. Participants in the location condition were instructed to memorize the locations of circles, while those in the orientation condition were asked to memorize the orientations of arrows. Both groups were explicitly instructed to disregard the shapes’ color. Each condition (location or orientation) comprised four blocks, with the avatar appearing on the left or right side of the screen for two blocks each. Each block included 40 trials, yielding a total of 320 formal trials in the experiment.

Besides the location or orientation trials, participants also underwent a filler session. In this session, participants were instructed to memorize both feature dimensions of the memory array. The filler session included four blocks of 40 trials each, leading to a total of 160 filler trials per participant. All other procedures remained consistent with Experiment 1.

## Results

We conducted a 2 × 2 mixed-measure ANOVA on the RTs of target-present trials with Target Dimension (Location vs. Orientation) as the between-participant variable and Irrelevant Feature (No change vs. Change) as the within-participant variable. The main effects of Target Dimension (*F* (1, 74) = 16.712, *p* < 0.001, η_p_^2^ = 0.184) and Irrelevant Feature (*F* (1, 74) = 36.291, *p* < 0.001, η_p_^2^ = 0.329), as well as their interaction (*F* (1, 74) = 4.291, *p* = 0.042, η_p_^2^ = 0.055), were all significant. Post-hoc pairwise comparisons revealed that changes in the irrelevant feature significantly slowed participants’ reaction speed both in the location (*p* = 0.007) and orientation (*p* < 0.001) conditions (see Fig. 2B). On the other hand, participants responded significantly faster in the location condition than in the orientation condition, regardless of changes to irrelevant features (*ps* < 0.001).

## Discussion

In Experiment 2, we observed a consistent encoding mechanism regardless of the target dimension when the irrelevant dimension exhibited high discriminability. Specifically, changes in the irrelevant feature consistently influenced participants’ target discrimination irrespective of the discriminability of the target dimension (location or orientation), indicating the presence of OBE in both conditions. Therefore, our findings suggest that the encoding mechanism employed under others’ perspectives depends on the attributes of the irrelevant dimension, with no decisive influence from the properties of the target dimension.

Interestingly, our findings revealed a significant interaction between the Target Dimension and the Irrelevant Feature, suggesting that the RT difference between the No Change and Change conditions was larger when the target dimension was orientation than when it was location. This finding indicates that while the target dimension does not dictate the encoding mechanism utilized by individuals, it may modulate the extent to which irrelevant changes interfere with target discrimination. Consequently, there is a third possibility for our results: the relative discriminability between the target and the irrelevant dimensions played a key role. Specifically, individuals exhibited OBE when the discriminability of the irrelevant dimension was higher than (Target _orientation_ & Irrelevant _location_, Target _orientation_ & Irrelevant _color_) or similar to (Target _location_ & Irrelevant _color_) that of the target dimension. When the irrelevant dimension had lower discriminability than the target dimension (Target _location_ & Irrelevant _orientation_), FBE predominated under others’ perspectives. We designed Experiment 3 to investigate this possibility.

### Experiment 3

The purpose of Experiment 3 was to investigate whether the encoding mechanism under others’ perspectives is solely influenced by the discriminability of the irrelevant dimension or by the relative discriminability between the target and irrelevant dimensions. In this experiment, both the target and irrelevant dimensions were manipulated to have low discriminability. Previous studies have demonstrated that a conjunction of different colors in one object can be perceived as a feature with low discriminability [19]. Therefore, we employed color conjunction as the irrelevant dimension and orientation as the target dimension to examine our hypotheses. If the encoding mechanism under others’ perspectives solely depends on the discriminability of the irrelevant dimension, we expect to observe an FBE effect as found in Experiment 1. However, if the encoding mechanism for adopting others’ perspectives is determined by the relative discriminability between two dimensions, we anticipate observing an OBE effect similar to that found in the location condition of Experiment 2.

## Methods

### Participants

The sample size for Experiment 3 was determined by a priori power analysis with G*Power 3. Based on a conservatively estimated medium effect size (*d* = 0.50), a power of 0.80, and an alpha level of 0.05, the power analysis conducted with a paired sample *t*-test (two-tailed) yielded a minimum sample size of 34. Ultimately, a total of 36 undergraduates were recruited for Experiment 3. Each participant received a compensation of 30 RMB for their involvement. After removing two participants whose RTs exceeded three standard deviations from the group mean, we analyzed data from the remaining 34 participants (15 males and 19 females, *M* = 19.735 years old, *SD* = 1.082).

### Stimuli

**Fig. 3:**
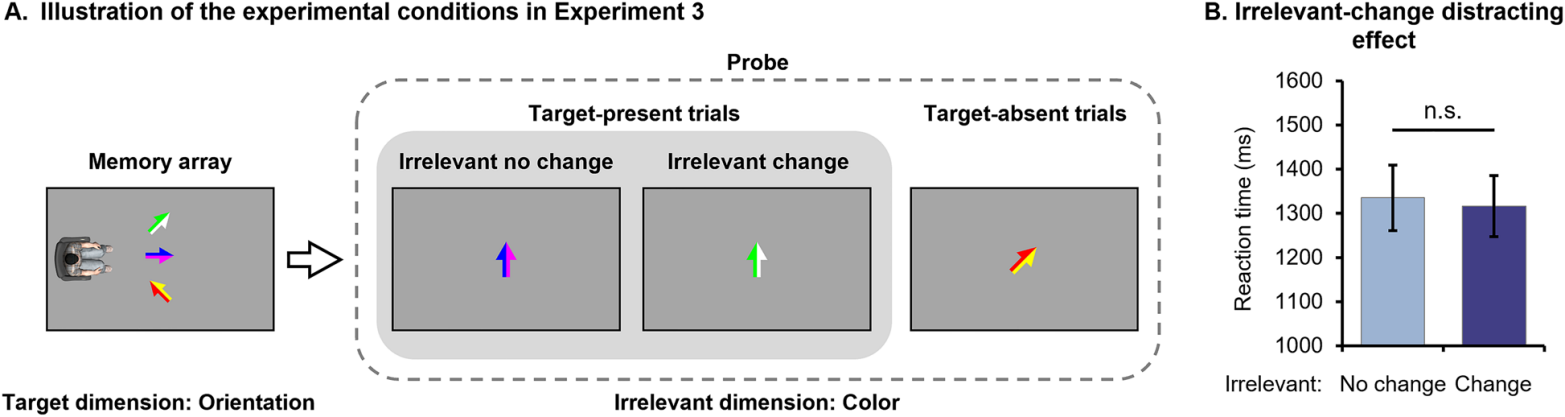
Illustration of experimental conditions (**A**) and results (**B**) for Experiment 3

The memory array included three arrows, each composed of two colors (see Fig. 3A). The color schemes of each arrow were randomly created by selecting any two of the six color values from Experiment 2’s color pool, while the orientations of the arrows were determined in a similar manner as in Experiment 1.

### Design and procedure

In Experiment 3, we utilized a within-participant design with Irrelevant Feature (No change vs. Change) as a single variable. Participants were instructed to adopt the avatar’s perspective to memorize the arrows’ orientations and ignore their colors. They then had to determine if the target feature of the probe arrow appeared in the previous memory array viewed from the avatar’s perspective. In the Irrelevant Feature “No change” condition, the probe arrow remained unchanged in both the target and irrelevant dimensions. In the “Change” condition, we employed a new pair of colors while keeping the orientation unchanged. All other procedures were identical to those described in the orientation condition of Experiment 2.

## Results

A paired-sample *t* test showed no significant difference in participants’ RTs between the No-change and Change conditions, *t* (33) = 0.525, *p* = 0.603, *Cohen’s d* = 0.090 (see Fig. 3B).

## Discussion

The results of Experiment 3 contradicted the relative discriminability hypothesis by showing that participants did not automatically encode irrelevant features when both the target and irrelevant dimensions were less discriminable. Therefore, we propose that the encoding mechanism under others’ perspectives is primarily determined by the discriminability of the irrelevant dimension, rather than the relative discriminability between the target and irrelevant dimensions.

### Combined analysis of experiments 1–3

Although Experiment 2 showed that the encoding mechanism under others’ perspectives was not influenced by the attributes of the target dimension, this conclusion is limited to situations where the irrelevant dimension exhibits high discriminability. Further investigation is needed to ascertain its applicability in low discriminability situations.

**Fig. 4:**
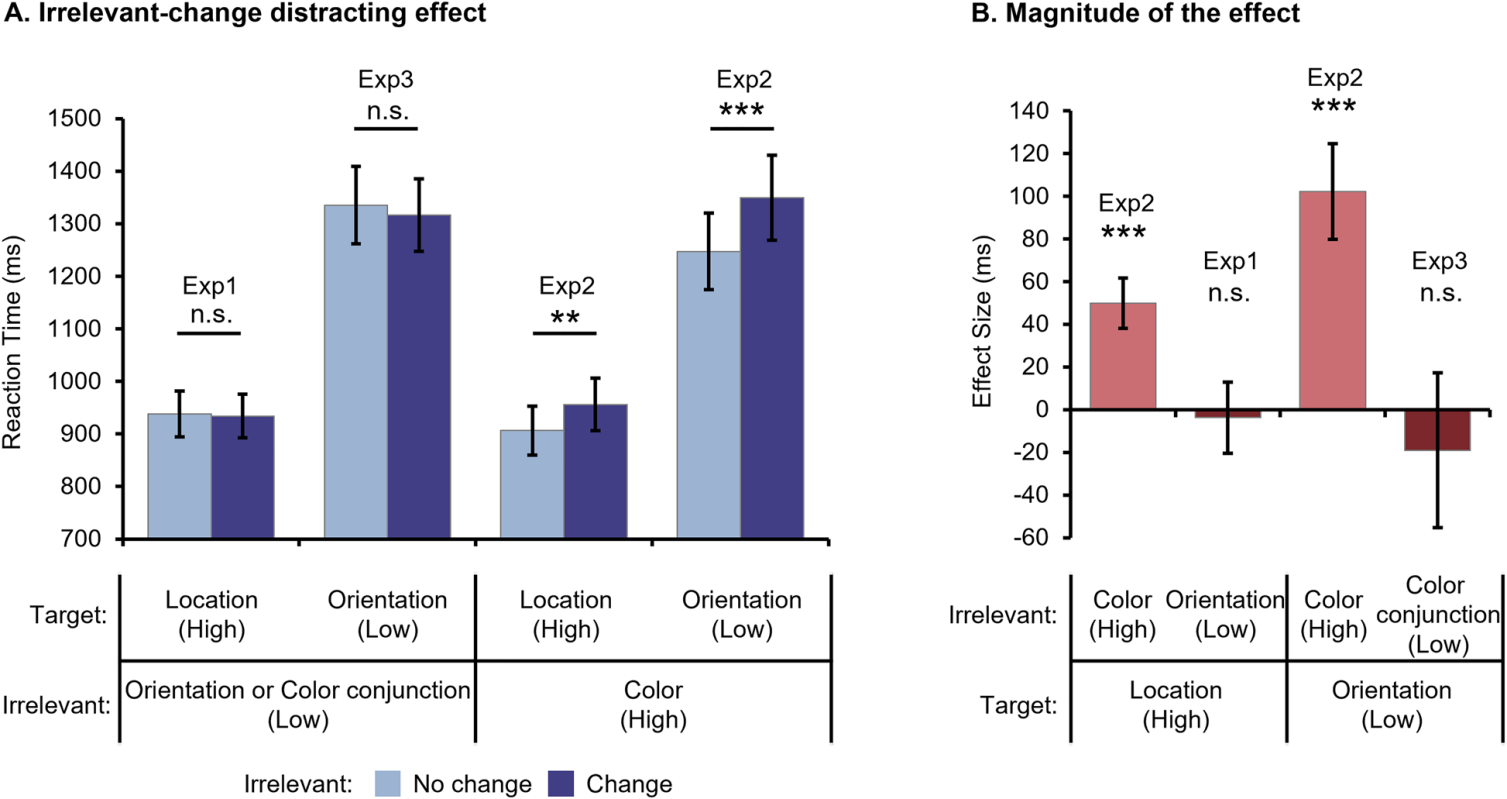
Participants’ RTs (**A**) and the magnitude of irrelevant change distracting effects (**B**) across different conditions in Experiment 1–3. While the left two panels in (A) were newly plotted, the right two panels of (A) corresponded to Fig. 2B and were included here to facilitate comparison

To clarify this issue, we selected two conditions characterized by less discriminable irrelevant dimensions for further comparison: the location condition of Experiment 1 (Irrelevant: Orientation) and the orientation condition of Experiment 3 (Irrelevant: Color-Conjunction). Subsequently, a 2 × 2 mixed-measure ANOVA was conducted on participants’ RTs to compare these two conditions, with Target Dimension (Location vs. Orientation) as the between-participant variable and Irrelevant Feature (No change vs. Change) as the within-participant variable. There was a significant main effect of Target Dimension,* F* (1, 76) = 25.481, *p* < 0.001, η_p_^2^ = 0.256. Participants responded significantly faster when they were recognizing the location rather than the orientation of the probe arrow (see Fig. 4A). However, the main effect of Irrelevant Feature (*F* (1, 76) = 0.370, *p* = 0.545, η_p_^2^ = 0.005) and the interaction (*F* (1, 75) = 0.166, *p* = 0.685, η_p_^2^ = 0.002) did not reach significance.

Then, we calculated the magnitude of the irrelevant-change distracting effect (Effect Size = RT _Change_ – RT _No change_) across three experiments to further assess the impact of the target and irrelevant dimensions. We selected four conditions with different levels of discriminability in the target and irrelevant dimensions: Target _high (location)_ & Irrelevant _high (color)_, Target _high (location)_ & Irrelevant _low (orientation)_, Target _low (orientation)_ & Irrelevant _high (color)_, and Target _low (orientation)_ & Irrelevant _low (color-conjunction)_ (see Fig. 4B). These conditions corresponded to the location condition of Experiment 2 and 1, as well as the orientation condition of Experiment 2 and 3, respectively. We aimed to examine whether the effect size was significantly greater than zero only for the high-discriminability irrelevant dimension, regardless of the discriminability of the target dimension. One-sample *t* tests showed that the effect size was significantly larger than zero in both the Target _high_ & Irrelevant _high_ (*t* (37) = 4.260, *p* < 0.001, *Cohen’s d* = 0.691) and the Target _low_ & Irrelevant _high_ conditions (*t* (37) = 4.569, *p* < 0.001, *Cohen’s d* = 0.741), but not in the Target _high_ & Irrelevant _low_ (*t* (41) = 0.226, *p* = 1, *Cohen’s d* = 0.035) or the Target _low_ & Irrelevant _low_ condition (*t* (33) = 0.525, *p* = 1, *Cohen’s d* = 0.090).

## Discussion

By controlling the discriminability of the irrelevant dimension, we found that altering the discriminability of the target dimension did not impact participants’ encoding mechanisms under others’ perspectives. The FBE was consistently employed in conditions with less discriminable irrelevant dimensions, while OBE was consistently utilized in conditions with highly discriminable ones, regardless of the attributes of the target dimension. Overall, these findings suggest that encoding mechanisms under others’ perspectives are primarily determined by the discriminability of the irrelevant dimension rather than that of the target one.

### Experiment 4

Our findings thus far have confirmed that individuals utilize a coarse-to-fine rule for encoding visual information in the VSPT task. However, it could be argued that participants may adopt an object-rotation strategy instead of performing mental-body transformation as instructed to complete the task. To address this issue, we included an object-rotation condition in Experiment 4 where participants were explicitly instructed to mentally rotate the visual stimuli from the avatar’s perspective into their own viewpoint to complete the task. Comparing this condition with a typical mental-body transformation condition (where participants were instructed to mentally transform their bodies to the avatar’s location and process visual stimuli from that perspective, as used in Experiment 1–3) will help clarify participants’ processing strategies. If participants employ the same strategy (object-rotation) in both conditions, there should be no discernible behavioral differences between them; otherwise, distinct encoding rules would contradict the argument.

**Fig. 5:**
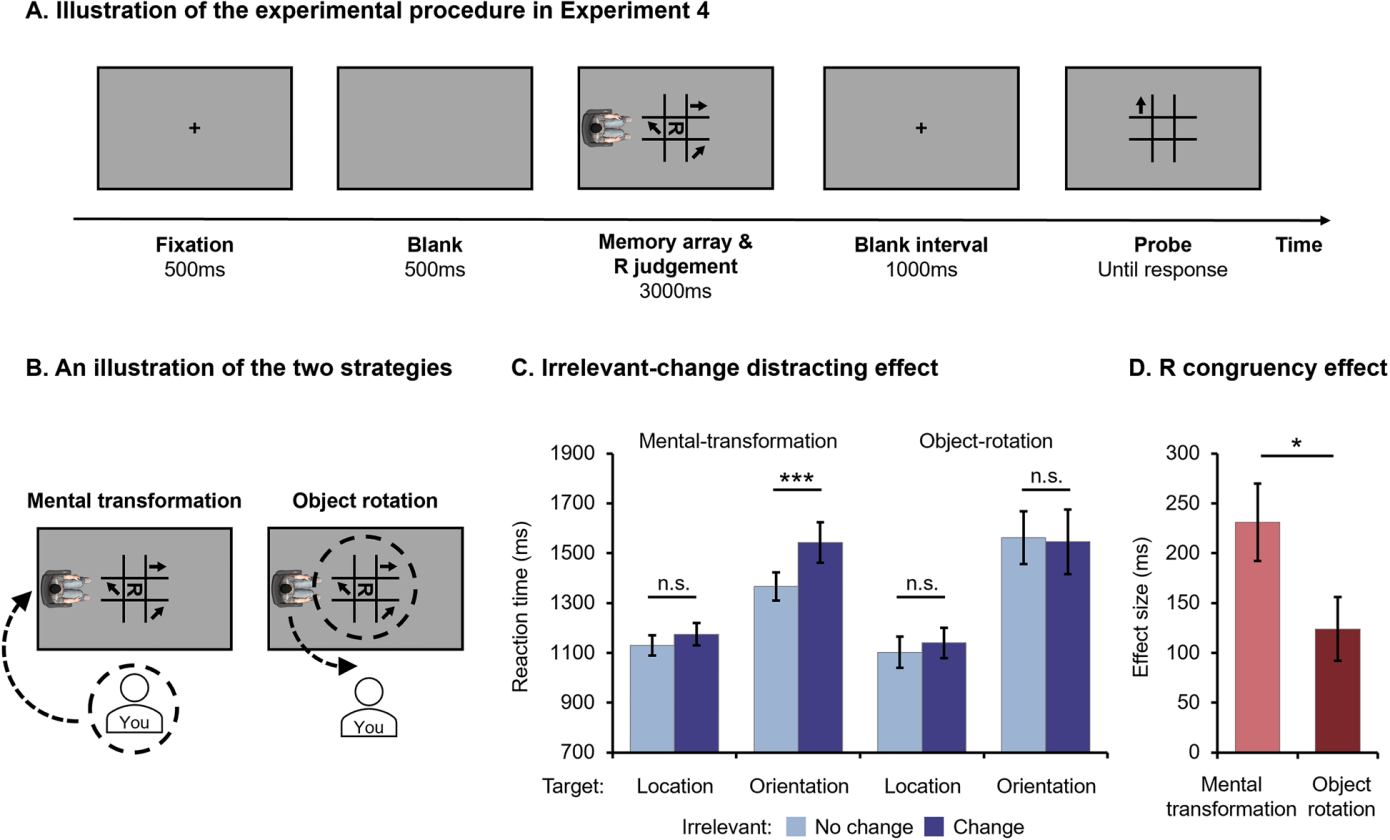
Illustration of the experimental procedure (**A**), conditions (**B**), and results (**C**&**D**) for Experiment 4. The dashed circles and arrows in (B) are exclusively employed for illustrative purposes to indicate the conceptual directions of mental-transformation or object-rotation and were not actually presented during the experiment

Additionally, we introduced a novel letter-discrimination task to further elucidate the distinction between the execution of the two strategies. Participants were instructed to determine whether an R, rotated 90° clockwise or counterclockwise and presented in the middle of the matrix (see Fig. 5A), was presented in its normal form (R) or as a mirror image (Я). If participants employ the same strategy (object-rotation) in both the mental-body transformation and the object-rotation conditions, there should be no observable behavioral differences on R discrimination between them. Otherwise, distinct performance on R discrimination would challenge this assumption.

## Methods

### Participants

The sample sizes of Experiment 4 were determined by a priori power analysis with MorePower 6.0.4. The result of Experiment 1 revealed a large effect size in feature encoding, leading us to estimate a standard large effect size (η_p_^2^ = 0.14). Based on this effect size, a power of 0.8, and an alpha level of 0.05, the power analysis conducted with a 2 × 2 × 2 mixed ANOVA produced a minimal sample size of 52 participants.

A total of 70 undergraduates participated in Experiment 4. All participants were provided with a compensation of 50 RMB for their participation. Data from two participants were excluded: one for outlier accuracy below three standard deviations from the mean of its respective group, and the other for recording error. Thus, data from the remaining 68 participants (31 males and 37 females, aged 18–22 years, *M* = 19.824, *SD* = 0.772) were included for subsequent analyses.

### Stimuli

A black letter R, rotated 90° clockwise or counterclockwise, was positioned at the center of the 3 × 3 matrix. The R could be either normal (R) or mirrored (Я). The other stimuli remained consistent as in Experiment 1.

### Design and procedure

The design of Experiment 4 was modified based on Experiment 1. In each trial, the avatar was randomly positioned on either the left or right side of the screen while facing the matrix. Additionally, a letter “R” appeared in the middle of the matrix along with the memory array (see Fig. 5A). Participants were randomly assigned to either the mental-transformation condition or the object-rotation condition (see Fig. 5B). Apart from the transformation/rotation instructions they received, all other settings were identical between the two conditions.

At the beginning of experiment, all participants were required to complete the identical memory task as in Experiment 1. Each participant underwent three sessions: the *location* and *orientation* session, along with a *both* session serving as filler trials. Thus, this experiment employed a 2 (Strategy: Mental-transformation vs. Object-rotation) × 2 (Target Dimension: Location vs. Orientation) × 2 (Irrelevant Feature: No change vs. Change) mixed design.

Besides, to further clarify the distinction between the implementation of the two strategies, participants were also instructed to complete the R discrimination task during the presentation of the memory array, as previously described. They had to discern whether the “R” was normal or mirrored as quickly and accurately as possible. Two congruency conditions were established based on the spatial relationship between R and the avatar. In some trials, R’s opening faced towards the avatar, which was classified as the congruent condition. Conversely, in other trials, R’s opening faced away from the avatar, constituting the incongruent condition. The magnitude of the congruency effect (Effect size = RT _incongruent_ – RT _congruent_) was used to estimate behavioral differences between the two strategy conditions.

To ensure consistent processing time for visual stimuli across participants, both the memory array and the letter R were presented for a duration of 3000 ms. Participants were explicitly instructed to finish their discrimination on the R and memorization of the arrows before they vanished from view. If no response was received within 3000 ms, a warning message would be displayed, and the trial would end without any further presentation of a probe stimulus.

Each participant completed 12 blocks (four in each session) of 20 trials each, resulting in a total of 160 formal trials and 80 filler trials. Within each block, the position of the avatar, the opening direction of R, and whether R is normal or mirrored were all randomized. All other aspects regarding design and procedure remained consistent with Experiment 1.

## Results

To investigate the impact of different strategies on encoding rules, we conducted a 2 × 2 × 2 mixed ANOVA on participants’ RTs in the memory task, with Strategy (Mental-transformation vs. Object-rotation) as a between-participant variable and Target Dimension (Location vs. Orientation) as well as Irrelevant Feature (No change vs. Change) as within-participant variables. The results showed significant main effects for Target Dimension (*F* (1, 66) = 43.579, *p* < 0.001, η_p_^2^ = 0.398) and Irrelevant Feature (*F* (1, 66) = 6.679, *p* = 0.012, η_p_^2^ = 0.092), as well as a significant interaction between Strategy and Irrelevant Feature, *F* (1, 66) = 4.568, *p* = 0.036, η_p_^2^ = 0.065. Importantly, the three-way interaction among Strategy, Target Dimension, and Irrelevant Feature was also significant, *F* (1, 66) = 4.742, *p* = 0.033, η_p_^2^ = 0.067. Post-hoc pairwise comparisons revealed that participants in the mental-transformation group responded significantly faster in the No change compared to the Change condition when the target dimension was orientation (*p* = 0.003), but not when it was location (*p* = 0.136, see Fig. 5C). However, there were no significant differences between the No change and Change conditions for participants in the object-rotation condition (*ps* > 0.1). No other main effects or interactions reached significance, either (*ps* > 0.1).

Then, we subjected the magnitude of the R congruency effect to an independent-sample *t* test between the two strategy conditions, which revealed a significantly larger effect size in the mental-transformation condition compared to the object-rotation condition, *t*(66) = 2.128, *p* = 0.037, Cohen’s *d* = 0.516 (see Fig. 5D).

## Discussion

Our findings revealed distinct encoding mechanisms employed when performing mental-transformation and object-rotation strategies. When participants utilize the mental-transformation strategy, a similar outcome to Experiment 1 was observed: highly discriminable irrelevant features were automatically encoded while less discriminable ones were not, indicating a “coarse-to-fine” mechanism at play. However, when instructed to adopt the object-rotation strategy, irrelevant features were consistently disregarded irrespective of their discriminability level, suggesting a consistent utilization of FBE. Moreover, we also observed varying strength in the R congruency effect between the mental-transformation condition and the object-rotation condition, suggesting distinct visual processing mechanisms in these two groups. Collectively, our findings provide evidence supporting the distinct nature of encoding mechanisms and cognitive processes involved in mental-body transformation and object-rotation tasks, thereby confirming that previous reported encoding mechanisms are specifically associated with mental-body transformation and embodied processing rather than an object-rotation strategy.

### General discussion

Understanding how objects are encoded and memorized from others’ perspectives is pivotal for comprehending and gaining insights into others’ visual worlds. However, despite an abundance of studies investigating self-perspective encoding of object features [1, 20, 23, 44, 56], the underlying mechanisms involved in object encoding while adopting another individual’s perspective remain unclear. In this study, we addressed this question for the first time by employing a modified change-detection task where participants adopted an avatar’s perspective to memorize features in a specific dimension of a given memory array. Across four experiments, we observed that individuals may employ either object-based encoding or feature-based encoding when adopting another person’s perspective, depending on the attributes of the task-irrelevant feature dimension. Specifically, if these irrelevant features exhibit high discriminability (e.g., location and color), they will be simultaneously extracted into memory from the same perspective along with the intentionally encoded target features. However, if these features possess low discriminability (e.g., orientation and color-conjunction), they will not be memorized. By independently manipulating changes in both irrelevant and target features, we have demonstrated that the encoding approaches were solely determined by attributes of the irrelevant dimension rather than those of the target dimension or differences in discriminability between them. Furthermore, our findings provide compelling evidence that these results indeed arose from other-perspective processing rather than self-perspective processing. Thus, our results reveal a “coarse-to-fine” rule governing memory encoding when adopting others’ perspectives. These findings significantly contribute to our understanding of how the human brain functions from multiple perspectives to encode and memorize the world, as well as to a deeper comprehension of how people understand the world through others’ eyes.

Considering the previously observed “coarse-to-fine” rule in self-perspective processing [18, 23], our findings suggest that there might exist similarities in the encoding mechanisms employed by the human brain during processing under other- and self-perspectives, aligning with the hypothesis of a self-other shared mechanism [28]. Moreover, we propose that embodied processing plays a crucial role in generating this object encoding that exhibits similarity across perspectives. When individuals engage in embodied processing, they undergo mental simulation of others’ sensations in their brains by utilizing their knowledge and experience, as if perceiving through the eyes of others [16, 17], thereby facilitating the formation of shared experiences. Conversely, if individuals do not adopt an embodied approach but instead use alternative strategies to complete the task, their encoding of visual stimuli will deviate from the “coarse-to-fine” rule. Therefore, embodied processing may serve as a crucial foundation for engaging in other- and self-perspective processing through similar mechanisms. However, the current conclusion is derived by a comparative analysis of our present study with previous research on self-perspective encoding. To further substantiate this finding, it is imperative for future studies to directly compare the encoding mechanisms involved in self- and other-perspective processing within a single experiment.

Notably, our findings have significantly broadened our understanding of perspective taking by revealing distinct memory encoding rules employed in diverse task strategies. Specifically, while numerous studies have demonstrated that participants typically rely on mental-body transformation to complete VSPT [32, 43], an alternative possibility exists where participants may employ an object-rotation strategy, therefore introducing a confounding explanation for the mechanism of the VSPT task. Our results show, for the first time, notable differences in encoding mechanisms employed when using these two strategies. When participants utilize the mental-transformation strategy, a “coarse-to-fine” mechanism is at play. However, when adopting the object-rotation strategy, there is consistent utilization of FBE. These results suggest that the embodied approach may offer distinct information about others’ perspectives compared to rotating objects, as it facilitates the encoding of irrelevant feature information into working memory—a process not observed during object-rotation. We propose that there are two possible explanations for these distinctions. First, as rotating target features alone is sufficient for task completion, it is likely that during object-rotation, only task-relevant target features of the memory array are rotated into self-perspective while task-irrelevant features are disregarded to save cognitive resource. Consequently, only the target features are memorized while irrelevant ones are not. However, it is also plausible that both features of the memory array are rotated into self-perspective during object rotation; nevertheless, due to participants needing to retain the complex rotated objects in memory and simultaneously process object features based on the maintained representations, this imposes a significant burden on cognitive resources. Therefore, to conserve cognitive resources and facilitate encoding of target information, our brain may restrict the encoding of irrelevant feature information. Nonetheless, our current study cannot differentiate between these two possibilities. Future studies should continue to investigate these hypotheses and further explore the underlying neural mechanisms associated with these distinct approaches.

Additionally, our study has further advanced the understanding of hierarchical organization in visual working memory. In self-perspective processing, individual features are typically integrated into objects to enhance memorization efficiency [13, 36, 57]. Our findings reveal that this hierarchical organization also applies to other-perspective processing of visual stimuli, wherein individual features are automatically aggregated into objects in an allocentric manner. These results highlight that hierarchical organization constitutes a foundational mechanism of visual working memory, functioning independently of the visual perspective employed. Future research could further investigate the neural substrates underlying both egocentric and allocentric hierarchical organizations, exploring the similarities and distinctions between these mechanisms.

Finally, it is noteworthy that despite the employment of similar encoding manners, potential disparities may exist in other facets between processing from an allocentric perspective and one’s own viewpoint. For example, embodied processing involves constructing allocentric visual images in imagination based on an individual’s sensory-motor experience and perceptual inputs, resembling memory reconstruction using current information and retrieved experiences [12, 30]. Consequently, it is possible that the generated allocentric representation during embodied processing may lack sufficient details, similar to reconstructed memories. Therefore, an allocentric representation constructed from another person’s perspective may not be as exact or as precise as a representation created from one’s own perspective. Future research could further explore similarities and differences between outcomes of other- and self-perspective processing, as well as the cognitive and neural mechanisms underlying these processes.

## Conclusion

The present study investigated the memory encoding mechanisms employed when adopting others’ perspectives. We found that the adoption of encoding strategies conforms to the “coarse-to-fine” rule: individuals employ object-based encoding when the task-irrelevant dimension of objects possesses high discriminability, and feature-based encoding when the irrelevant dimension has low discriminability. These mechanisms are specific to embodied VSPT, and using alternative strategies for VSPT tasks will lead to different encoding rules. Considering the spontaneous nature of perspective taking in daily life, future research could explore the encoding mechanisms underlying implicit VSPT, investigating both its similarities and differences with explicit adoption of others’ visual perspectives.

## Supplementary Information

Supplementary Material 1. This file includes the ACC results as well as the analyses of participants’ egocentric errors across Experiments 1-4.

## Data Availability

All data, analysis scripts, and study materials are available at Open Science Framework, https://osf.io/3u6h4/?view_only = 9eb9e208f5344891a79ddfc27d6227bd.

## References

[1] Bichot NP, Heard MT, DeGennaro EM, Desimone R. A source for feature-based attention in the prefrontal cortex. Neuron. 2015;88(4):832–44. 10.1016/j.neuron.2015.10.001. 26526392 10.1016/j.neuron.2015.10.001 PMC4655197

[2] Blatt B, LeLacheur SF, Galinsky AD, Simmens SJ, Greenberg L. Does perspective-taking increase patient satisfaction in medical encounters? Acad Med. 2010;85(9):1445–52. 10.1097/ACM.0b013e3181eae5ec. 20736672 10.1097/ACM.0b013e3181eae5ec

[3] Brozzoli C, Gentile G, Bergouignan L, Ehrsson HH. A Shared representation of the space near oneself and others in the human premotor cortex. Curr Biol. 2013;23(18):1764–8. 10.1016/j.cub.2013.07.004. 24012310 10.1016/j.cub.2013.07.004

[4] Cavallo A, Ansuini C, Capozzi F, Tversky B, Becchio C. When far becomes near:perspective taking induces social remapping of spatial relations. Psychol Sci. 2017;28(1):69–79. 10.1177/0956797616672464. 27864372 10.1177/0956797616672464

[5] Charness G, Gneezy U, Kuhn MA. Experimental methods: between-subject and within-subject design. J Econ Behav Organ. 2012;81(1):1–8. 10.1016/j.jebo.2011.08.009.

[6] Cole GG, Millett AC. The closing of the theory of mind: a critique of perspective-taking. Psychon Bull Rev. 2019;26(6):1787–802. 10.3758/s13423-019-01657-y. 31515733 10.3758/s13423-019-01657-y

[7] Deroualle D, Borel L, Tanguy B, Bernard-Demanze L, Devèze A, Montava M, Lavieille J-P, Lopez C. Unilateral vestibular deafferentation impairs embodied spatial cognition. J Neurol. 2019;266(1):149–59. 10.1007/s00415-019-09433-7. 31230115 10.1007/s00415-019-09433-7

[8] Decety J, Chaminade T. When the self represents the other: A new cognitive neuroscience view on psychological identification. Conscious Cogn. 2003;12(4):577–96. 10.1016/S1053-8100(03)00076-X. 14656502 10.1016/s1053-8100(03)00076-x

[9] Decety J, Sommerville JA. Shared representations between self and other: a social cognitive neuroscience view. Trends Cogn Sci. 2003;7(12):527–33. 10.1016/j.tics.2003.10.004. 14643368 10.1016/j.tics.2003.10.004

[10] Erle TM, Topolinski S. The grounded nature of psychological perspective-taking. J Pers Soc Psychol. 2017;112(5):683–95. 10.1037/pspa0000081. 28253002 10.1037/pspa0000081

[11] Faul F, Erdfelder E, Buchner A, Lang AG. Statistical power analyses using G*Power 3.1: tests for correlation and regression analyses. Behav Res Methods. 2009;41(4):1149–60. 10.3758/BRM.41.4.1149. 19897823 10.3758/BRM.41.4.1149

[12] Fivush R, Grysman A. Accuracy and reconstruction in autobiographical memory: (Re)consolidating neuroscience and sociocultural developmental approaches. WIREs Cognit Sci. 2023;14(3):e1620. 10.1002/wcs.1620. 10.1002/wcs.1620 36125799

[13] Fan Y, Wang M, Fang F, Ding N, Luo H. Two-dimensional neural geometry underpins hierarchical organization of sequence in human working memory. Nat Hum Behav. 2025;9(2):360–75. 10.1038/s41562-024-02047-8. 39511344 10.1038/s41562-024-02047-8

[14] Galinsky AD, Ku G, Wang CS. Perspective-taking and self-other overlap: fostering social bonds and facilitating social coordination. Group Process Intergroup Relat. 2005;8(2):109–24. 10.1177/1368430205051060.

[15] Galinsky AD, Maddux WW, Gilin D, White JB. Why It Pays to get inside the head of your opponent:the differential effects of perspective taking and empathy in negotiations. Psychol Sci. 2008;19(4):378–84. 10.1111/j.1467-9280.2008.02096.x. 18399891 10.1111/j.1467-9280.2008.02096.x

[16] Gallese V. Bodily selves in relation: embodied simulation as second-person perspective on intersubjectivity. Philosoph Transact Royal Soc B Biolog Sci. 2014;369(1644):20130177. 10.1098/rstb.2013.0177. 10.1098/rstb.2013.0177 PMC4006180 24778374

[17] Gallese V, Rochat M, Cossu G, Sinigaglia C. Motor cognition and its role in the phylogeny and ontogeny of action understanding. Dev Psychol. 2009;45(1):103. 10.1037/a0014436. 19209994 10.1037/a0014436

[18] Gao Z, Ding X, Yang T, Liang J, Shui R. Coarse-to-fine construction for high-resolution representation in visual working memory. PLoS ONE. 2013;8(2):e57913. 10.1371/journal.pone.0057913. 23469103 10.1371/journal.pone.0057913 PMC3585254

[19] Gao Z, Li J, Yin J, Shen M. Dissociated mechanisms of extracting perceptual information into visual working memory. PloS One. 2010;5(12):e14273. 10.1371/journal.pone.0014273. 21170315 10.1371/journal.pone.0014273 PMC3000807

[20] Gao Z, Yu S, Zhu C, Shui R, Weng X, Li P, Shen M. Object-based encoding in visual working memory: evidence from memory-driven attentional capture. Sci Rep. 2016;6(1):22822. 10.1038/srep22822. 26956084 10.1038/srep22822 PMC4783775

[21] Gardner MR, Potts R. Hand dominance influences the processing of observed bodies. Brain Cogn. 2010;73(1):35–40. 10.1016/j.bandc.2010.02.002. 20338681 10.1016/j.bandc.2010.02.002

[22] Grizenko N, Zappitelli M, Langevin J-P, Hrychko S, El-Messidi A, Kaminester D, Pawliuk N, Stepanian MT. Effectiveness of a social skills training program using self/other perspective-taking: a nine-month follow-up. Am J Orthopsychiatry. 2000;70(4):501–9. 10.1037/h0087662. 11086528 10.1037/h0087662

[23] Gu Q, Dai A, Ye T, Huang B, Lu X, Shen M, Gao Z. Object-based encoding in visual working memory: a critical revisit. Quart J Exper Psychol. 2022;75(8):1397–410. 10.1177/17470218211052502. 10.1177/17470218211052502 34609217

[24] Ishida H, Suzuki K, Grandi LC. Predictive coding accounts of shared representations in parieto-insular networks. Neuropsychologia. 2015;70:442–54. 10.1016/j.neuropsychologia.2014.10.020. 25447372 10.1016/j.neuropsychologia.2014.10.020

[25] Kampis D, Southgate V. Altercentric cognition: how others influence our cognitive processing. Trends Cogn Sci. 2020;24(11):945–59. 10.1016/j.tics.2020.09.003. 32981846 10.1016/j.tics.2020.09.003

[26] Kessler K, Rutherford H. The two forms of visuo-spatial perspective taking are differently embodied and subserve different spatial prepositions. Front Psychol. 2010;1. 10.3389/fpsyg.2010.00213. 10.3389/fpsyg.2010.00213 PMC3153818 21833268

[27] Kessler K, Thomson LA. The embodied nature of spatial perspective taking: Embodied transformation versus sensorimotor interference. Cognition. 2010;114(1):72–88. 10.1016/j.cognition.2009.08.015. 19782971 10.1016/j.cognition.2009.08.015

[28] Keysers C, Gazzola V. Towards a unifying neural theory of social cognition. Prog Brain Res. 2006;156:379–401. 10.1016/S0079-6123(06)56021-2. 17015092 10.1016/S0079-6123(06)56021-2

[29] Kuhn G, Vacaityte I, D’Souza ADC, Millett AC, Cole GG. Mental states modulate gaze following, but not automatically. Cognition. 2018;180:1–9. 10.1016/j.cognition.2018.05.020. 29981964 10.1016/j.cognition.2018.05.020

[30] Loftus EF, Palmer JC. Reconstruction of automobile destruction: an example of the interaction between language and memory. J Verbal Learn Verbal Behav. 1974;13(5):585–9. 10.1016/S0022-5371(74)80011-3.

[31] Luck SJ, Vogel EK. The capacity of visual working memory for features and conjunctions. Nature. 1997;390(6657):279–81. 10.1038/36846. 9384378 10.1038/36846

[32] Martin AK, Kessler K, Cooke S, Huang J, Meinzer M. The right temporoparietal junction is causally associated with embodied perspective-taking. J Neurosci. 2020;40(15):3089–95. 10.1523/jneurosci.2637-19.2020. 32132264 10.1523/JNEUROSCI.2637-19.2020 PMC7141886

[33] Muraki EJ, Speed LJ, Pexman PM. Insights into embodied cognition and mental imagery from aphantasia. Nat Rev Psychol. 2023;2(10):591–605. 10.1038/s44159-023-00221-9.

[34] Michelon P, Zacks JM. Two kinds of visual perspective taking. Percept Psychoph. 2006;68(2):327–37. 10.3758/BF03193680. 10.3758/bf03193680 16773904

[35] Nurmsoo E, Bloom P. Preschoolers’ perspective taking in word learning:do they blindly follow eye gaze? Psychol Sci. 2008;19(3):211–5. 10.1111/j.1467-9280.2008.02069.x. 18315790 10.1111/j.1467-9280.2008.02069.x

[36] Nie Q-Y, Müller HJ, Conci M. Hierarchical organization in visual working memory: from global ensemble to individual object structure. Cognition. 2017;159:85–96. 10.1016/j.cognition.2016.11.009. 27914301 10.1016/j.cognition.2016.11.009

[37] Rainer G, Asaad WF, Miller EK. Selective representation of relevant information by neurons in the primate prefrontal cortex. Nature. 1998;393(6685):577–9. 10.1038/31235. 9634233 10.1038/31235

[38] Ratcliff R, Smith P. Perceptual discrimination in static and dynamic noise: the temporal relation between perceptual encoding and decision making. J Exp Psychol Gen. 2010;139:70–94. 10.1037/a0018128. 20121313 10.1037/a0018128 PMC2854493

[39] Samuel S, Hagspiel K, Eacott MJ, Cole GG. Visual perspective-taking and image-like representations: we don’t see it. Cognition. 2021;210:104607. 10.1016/j.cognition.2021.104607. 33508578 10.1016/j.cognition.2021.104607

[40] Samuel S, Legg EW, Manchester C, Lurz R, Clayton NS. Where was I? Taking alternative visual perspectives can make us (briefly) misplace our own. Quart J Exper Psychol. 2020;73(3):468–77. 10.1177/1747021819881097. 10.1177/1747021819881097 31544626

[41] Serences JT, Ester EF, Vogel EK, Awh E. Stimulus-specific delay activity in human primary visual cortex. Psychol Sci. 2009;20(2):207–14. 10.1111/j.1467-9280.2009.02276.x. 19170936 10.1111/j.1467-9280.2009.02276.x PMC2875116

[42] Sereno AB, Amador SC. Attention and memory-related responses of neurons in the lateral intraparietal area during spatial and shape-delayed match-to-sample tasks. J Neurophysiol. 2006;95(2):1078–98. 10.1152/jn.00431.2005. 16221750 10.1152/jn.00431.2005

[43] Seymour RA, Wang H, Rippon G, Kessler K. Oscillatory networks of high-level mental alignment: a perspective-taking MEG study. Neuroimage. 2018;177:98–107. 10.1016/j.neuroimage.2018.05.016. 29746907 10.1016/j.neuroimage.2018.05.016

[44] Shen M, Huang X, Gao Z. Object-based attention underlies the rehearsal of feature binding in visual working memory. J Exp Psychol Hum Percept Perform. 2015;41(2):479. 10.1037/xhp0000018. 25602968 10.1037/xhp0000018

[45] Shen M, Tang N, Wu F, Shui R, Gao Z. Robust object-based encoding in visual working memory. J Vis. 2013;13(2):1–1. 10.1167/13.2.1. 23378130 10.1167/13.2.1

[46] Sternberg S. The discovery of processing stages: extensions of donders’ method. Acta Physiol (Oxf). 1969;30:276–315. 10.1016/0001-6918(69)90055-9.

[47] Summerfield C, Egner T. Feature-based attention and feature-based expectation. Trends Cogn Sci. 2016;20(6):401–4. 10.1016/j.tics.2016.03.008. 27079632 10.1016/j.tics.2016.03.008 PMC4875850

[48] Surtees A, Apperly I, Samson D. The use of embodied self-rotation for visual and spatial perspective-taking. Front Human Neurosci. 2013;7(698). 10.3389/fnhum.2013.00698. 10.3389/fnhum.2013.00698 PMC3817588 24204334

[49] Trötschel R, Hüffmeier J, Loschelder DD, Schwartz K, Gollwitzer PM. Perspective taking as a means to overcome motivational barriers in negotiations: When putting oneself into the opponent’s shoes helps to walk toward agreements. J Pers Soc Psychol. 2011;101(4):771–90. 10.1037/a0023801. 21728447 10.1037/a0023801

[50] Vogel EK, McCollough AW, Machizawa MG. Neural measures reveal individual differences in controlling access to working memory. Nature. 2005;438(7067):500–3. 10.1038/nature04171. 16306992 10.1038/nature04171

[51] Vogel EK, Woodman GF, Luck SJ. Storage of features, conjunctions, and objects in visual working memory. J Exp Psychol Hum Percept Perform. 2001;27(1):92–114. 10.1037/0096-1523.27.1.92. 11248943 10.1037//0096-1523.27.1.92

[52] Wang M, Nie Q-Y. A computational account of conflict processing during mental imagery. Cogn Affect Behav Neurosci. 2024;24(5):816–38. 10.3758/s13415-024-01201-z. 39085587 10.3758/s13415-024-01201-z

[53] Ward E, Ganis G, Bach P. spontaneous vicarious perception of the content of another’s visual perspective. Curr Biol. 2019;29(5):874-880.e874. 10.1016/j.cub.2019.01.046. 30799242 10.1016/j.cub.2019.01.046

[54] Ward E, Ganis G, McDonough KL, Bach P. Perspective taking as virtual navigation? Perceptual simulation of what others see reflects their location in space but not their gaze. Cognition. 2020;199:104241. 10.1016/j.cognition.2020.104241. 32105910 10.1016/j.cognition.2020.104241

[55] Woodman GF. Spatial location is filtered out of visual working memory representations when task irrelevant, just like other features. Atten Percept Psychophys. 2021;83(4):1391–6. 10.3758/s13414-021-02263-8. 33728509 10.3758/s13414-021-02263-8

[56] Woodman GF, Vogel EK. Selective storage and maintenance of an object’s features in visual working memory. Psychon Bull Rev. 2008;15(1):223–9. 10.3758/PBR.15.1.223. 18605507 10.3758/pbr.15.1.223

[57] Xu Y. Encoding color and shape from different parts of an object in visual short-term memory. Percept Psychophys. 2002;64(8):1260–80. 10.3758/BF03194770. 12519024 10.3758/bf03194770

[58] Xu Y. The neural fate of task-irrelevant features in object-based processing. J Neurosci. 2010;30(42):14020–8. 10.1523/jneurosci.3011-10.2010. 20962223 10.1523/JNEUROSCI.3011-10.2010 PMC6634783

[59] Yin J, Zhou J, Xu H, Liang J, Gao Z, Shen M. Does high memory load kick task-irrelevant information out of visual working memory? Psychon Bull Rev. 2012;19(2):218–24. 10.3758/s13423-011-0201-y. 22215468 10.3758/s13423-011-0201-y

[60] Yuan X, Wang N, Geng H, Zhang S. Mentalizing another's visual world—a novel exploration via motion aftereffect. Front Psycholo. 2017;8. 10.3389/fpsyg.2017.01535. 10.3389/fpsyg.2017.01535 PMC5594217 28936191

[61] Zhang Q, Shen M, Tang N, Zhao G, Gao Z. Object-based encoding in visual working memory: a life span study. J Vis. 2013;13(10):11–11. 10.1167/13.10.11. 23962736 10.1167/13.10.11

