# Supplementary material for: How the brain memorizes the world from others’ perspectives: investigating allocentric encoding of object features during perspective taking
Source: BMC Psychol. 2025 Jul 1;13:691. doi: 10.1186/s40359-025-03022-2 (PMC12219340; doi:10.1186/s40359-025-03022-2)
Supplement: Supplementary file 1 — Supplementary Material 1. This file includes the ACC results as well as the analyses of participants’ egocentric errors across Experiments 1-4. [file 40359_2025_3022_MOESM1_ESM.docx]

**Supplementary Information for**

**How the brain memorizes the world from others’ perspectives: investigating allocentric encoding of object features during perspective-taking**

**This file includes:**

**Supplementary results for Experiments 1-4**

**Supplementary Figures S1-S4**

**Supplementary Tables S1**

**Experiment 1**

**Results**

*ACC Results*

The same two-way ANOVA conducted on participants’ ACCs also revealed two significant main effects and a significant interaction, *F* _target_ (1, 41) = 63.838, *p* < .001, η_p_^2^ = .609; *F* _irrelevant_ (1, 41) = 6.852, *p* = .012, η_p_^2^ = .143; *F* _interaction_ (1, 41) = 16.484, *p* < .001, η_p_^2^ = .287. Changes in the irrelevant feature significantly impaired participants’ accuracy in recognizing the orientation of the probe arrow (*p* < .001), but not in recognizing its location (*p* = .307, see Fig. S1). Meanwhile, participants exhibited significantly higher accuracy rates when recognizing the location rather than the orientation of the probe arrow, regardless of changes in irrelevant features (*ps* < .001).

**
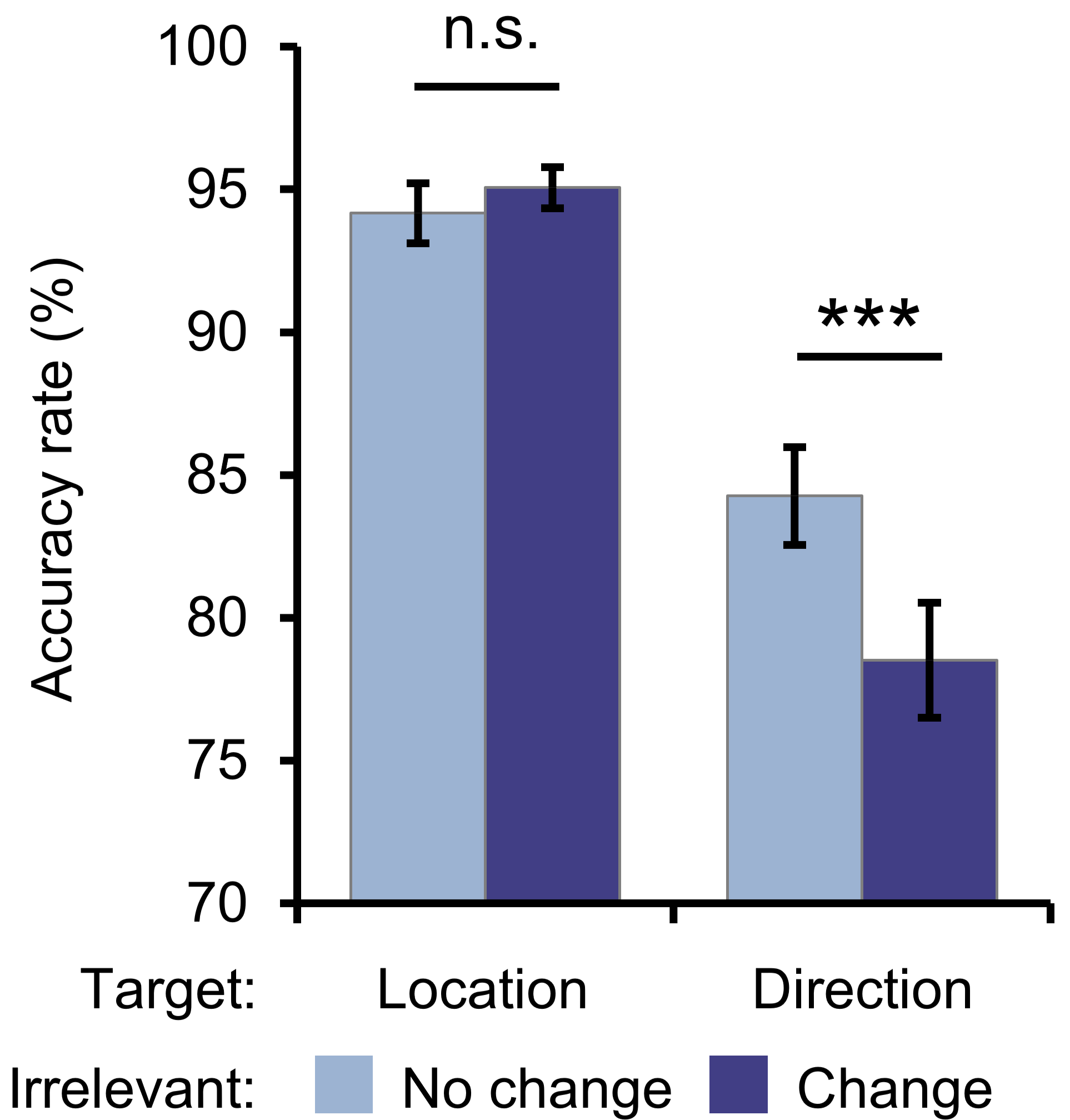
**

**Fig. S1** Participants’ ACCs across different conditions in the target-present trials in Experiment 1. Error bars represent one standard error, asterisks indicate significant pairwise comparisons, **p* < .05, ***p* < .01, ****p* < .001. “†” indicates a marginal significance. “n.s.” indicates the lack of statistical significance. These notations apply to all figures included in this Supplementary Material.

Table S1. Accuracies in Experiment 1-4 [*M*(*SE*)]

| Experiment | Target Dimension | Irrelevant Feature | |
| --- | --- | --- | --- |
|  |  | No change | Change |
| 1 | Location | 94.167% (1.049%) | 95.060% (0.725%) |
|  | Orientation | 84.226% (1.709%) | 78.512% (2.013%) |
| 2 | Location | 94.803% (0.785%) | 94.145% (0.930%) |
|  | Orientation | 89.145% (1.311%) | 84.342% (1.475%) |
| 3 | Orientation | 85.809% (1.867%) | 83.456% (2.705%) |
| 4 |  | Mental-transformation condition | |
|  | Location | 86.324% (1.868%) | 86.765% (1.933%) |
|  | Orientation | 80.883% (2.598%) | 67.353% (4.343%) |
|  |  | Object-rotation condition | |
|  | Location | 87.941% (2.265%) | 86.029% (2.893%) |
|  | Orientation | 79.118% (2.657%) | 75.294% (2.711%) |

*Egocentric Bias*

Since individuals often spontaneously process visual stimuli from their own perspectives when adopting others’ viewpoints (Surtees, Butterfill, & Apperly, 2012), we also investigated the existence of egocentric processing during allocentric feature encoding. Specifically, we examined participants’ false responses in situations where the target feature of the probe stimulus is absent from the avatar’s perspective but present in participants’ self-perspective within the memory array (indicating their misidentification of self-perspective features as other-perspective features). This exploration aimed to ascertain both the presence and frequency of egocentric bias, thereby promoting a comprehensive understanding of the mechanisms involved in allocentric feature encoding.

In 1.637% (*SD* = 1.531%) and 7.738% (*SD* = 3.785%) of the trials under the location and orientation condition, respectively, participants mistakenly identified target features that they had previously seen from their own perspectives in the memory array as appearing from the avatar’s perspective. These errors accounted for 29.613% (*SD* = 23.893%) and 48.349% (*SD* = 12.829%) of the total errors in the two conditions. The results of two paired sample *t*-tests showed that participants made more egocentric errors when recognizing the orientation rather than the location of the probe arrow, *t*(41) = 10.454, *p* < .001, Cohen’s *d* = 1.613. Additionally, there was a higher proportion of these egocentric errors to the total error in their respective conditions when recognizing the orientation as opposite to the location of the probe arrow, *t*(41) = 4.475, *p* < .001, Cohen’s *d* = .691.

Our findings revealed the existence of egocentric bias during individuals’ processing from others’ perspectives. Specifically, in certain trials, participants mistakenly perceived the target feature of the probe stimulus as having appeared in the preceding memory array from the avatar’s perspective, whereas it had actually emerged from their own perspectives. This discovery implies that individuals implicitly encoded and retained features based on their own perspectives while explicitly processing features from others’ viewpoints. Additionally, the egocentric bias is more pronounced when memorizing orientation rather than location, indicating that the magnitude of egocentric bias is task-dependent. The interference from self-perspective will be greater when the target feature is less discriminable and memory is challenging, compared to situations where the target feature is highly discriminable and the task is relatively easy.

**Experiment 2**

**Results**

*ACC Results*

Participants’ accuracy rates in target-present and target-absent trials were 90.609% (*SD* = 7.418%) and 92.237% (*SD* = 8.076%), separately. Overall, participants achieved 91.423% accuracy (*SD* = 7.024%).

We conducted a 2×2 mixed-measure ANOVA on the ACCs of target-present trials with Target Dimension (Location vs. Orientation) as the between-participant variable and Irrelevant Feature (No change vs. Change) as the within-participant variable. The main effects of the two variables, as well as their interactions, were all significant, *F* _target_ (1, 74) = 28.089, *p* < .001, η_p_^2^ = .275; *F* _irrelevant_ (1, 74) = 13.305, *p* < .001, η_p_^2^ = .152; *F* _interaction_ (1, 74) = 7.666, *p* = .007, η_p_^2^ = .094. A simple main effect analysis revealed that changes in the irrelevant feature significantly impaired participants’ accuracy in recognizing the orientation of the probe arrow (*p* < .001), but not in recognizing its location (*p* = .536, see Fig. S2 & Table S1). Meanwhile, participants exhibited significantly higher accuracy rates when recognizing the location rather than the orientation of the probe arrow, regardless of changes in irrelevant features (*ps* < .001).


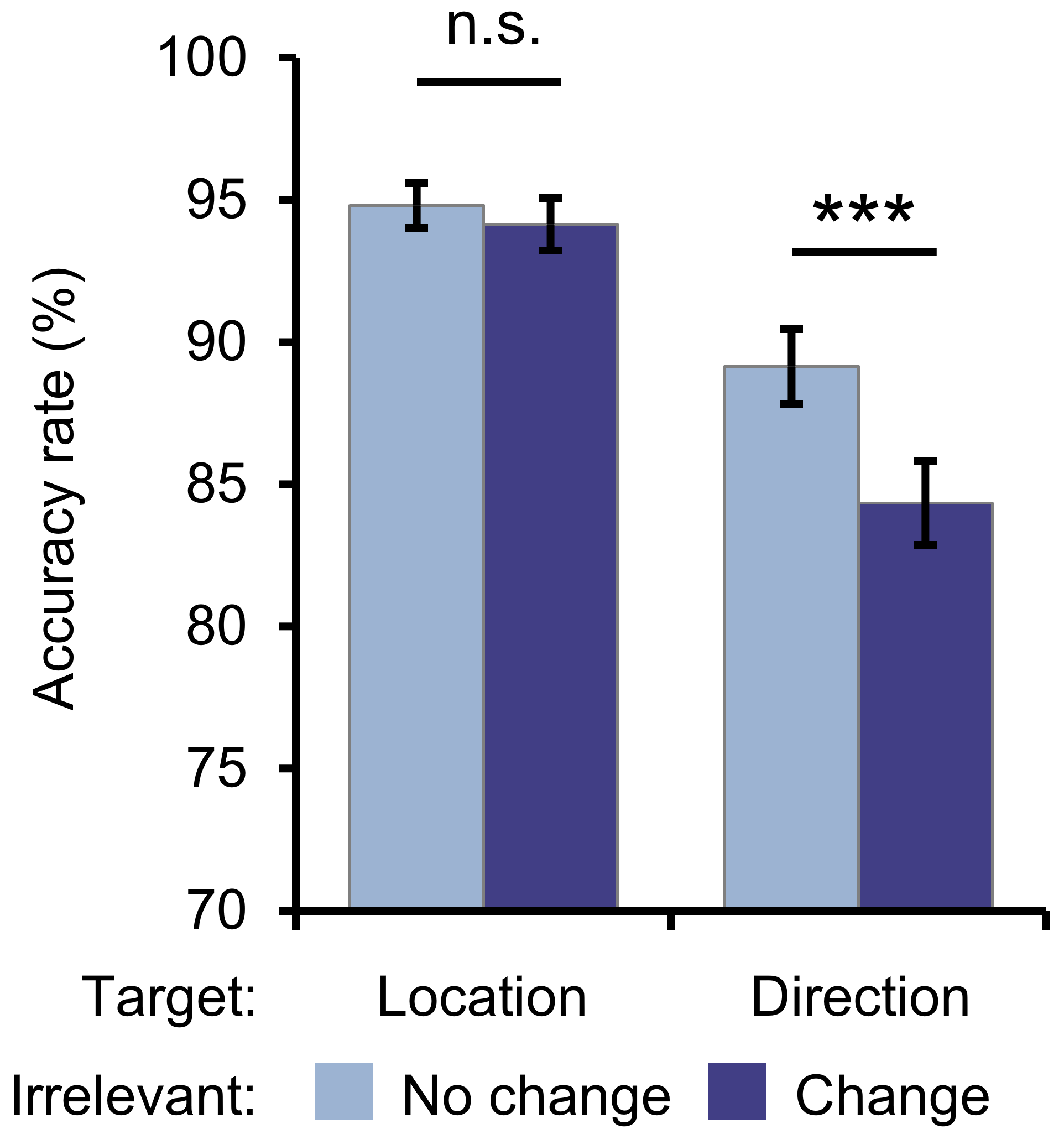


**Fig. S2** *Participants’ ACCs across different conditions in the target-present trials in Experiment 2.*

*Egocentric bias*

In 1.793% (*SD* = 1.645%) and 5.263% (*SD* = 3.698%) of the trials under the location and orientation condition, respectively, participants mistakenly identified target features that they had previously seen from their own perspectives in the memory array as appearing from the avatar’s perspective. These errors accounted for 31.701% (*SD* = 22.491%) and 44.398% (*SD* = 17.736%) of the total errors in the two conditions. The results of two independent sample *t*-tests showed that participants made more egocentric errors when recognizing the orientation rather than the location of the probe arrow, *t*(51.093) = 5.286, *p* < .001, Cohen’s *d* = 1.200. Additionally, there was a higher proportion of these egocentric errors to the total error in their respective conditions when recognizing the orientation as opposite to the location of the probe arrow, *t*(74) = 2.733, *p* = .008, Cohen’s *d* = .627.

**Experiment 3**

**Results**

*ACC Results*

Participants’ accuracy rates in target-present and target-absent trials were 84.632% (*SD* = 12.626%) and 87.426% (*SD* = 9.770%), separately. Overall, participants achieved 86.029% accuracy (*SD* = 9.798%).

A paired-sample *t* test showed no significant difference in participants’ ACCs between the No-change and Change conditions, *t* (33) = 1.393, *p* = .173, *Cohen’s* *d* = .239 (see Fig. S3).


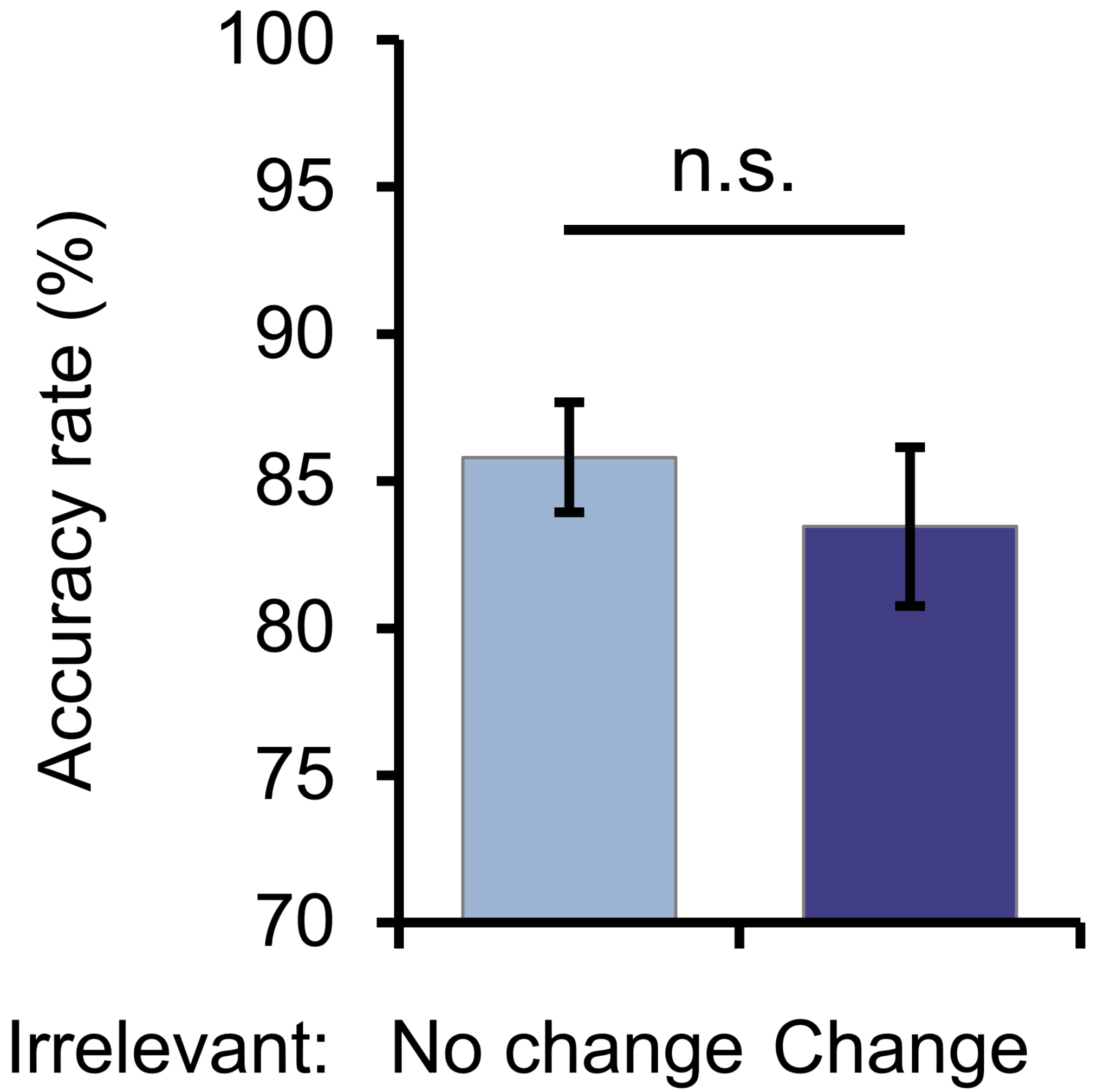


**Fig. S3** *Participants’ ACCs across different conditions in the target-present trials in Experiment 3.*

*Egocentric bias*

In 6.048% (*SD* = 4.053%) of the trials, participants mistakenly identified target features that they had previously seen from their own perspectives in the memory array as appearing from the avatar’s perspective. These errors accounted for 45.344% (*SD* = 13.684%) of the total errors.

**Experiment 4**

**Results**

*ACC Results*

Participants failed to make the R judgment within 3 seconds in 1.921% (*SD* = 2.503%) of the trials. Participants’ average accuracy rate on R judgement was 90.239% (*SD* = 10.632%).

Participants’ accuracy rates on probe judgement in target-present and target-absent trials were 81.213% (*SD* = 11.772%) and 82.592% (*SD* = 11.934%), separately. Overall, participants achieved 81.903% accuracy (*SD* = 9.885%).

To explore the impact of different strategies on the encoding mechanisms employed in the VSPT task, we conducted a 2×2×2 mixed ANOVA on participants’ ACCs in the memory task, with Strategy (Mental-transformation vs. Object-rotation) as a between-participant variable and Target Dimension (Location vs. Orientation) as well as Irrelevant Feature (No change vs. Change) as within-participant variables. We found that the main effects of Target Dimension and Irrelevant Feature were both significant, together with a significant interaction between them, *F* _target_ (1, 66) = 35.785, *p* < .001, η_p_^2^ = .352; *F* _irrelevant_ (1, 66) = 9.244, *p* = .003, η_p_^2^ = .123; *F* _target-irrelevant interaction_ (1, 66) = 14.088, *p* < .001, η_p_^2^ = .176. Importantly, the three-way interaction among Strategy, Target Dimension, and Irrelevant Feature was also significant, *F* (1, 66) = 8.121, *p* = .006, η_p_^2^ = .110. A simple main effect analysis revealed that participants in the mental-transformation condition showed significantly higher ACCs in the No change compared to the Change condition when the target dimension was orientation (*p* < .001), but not when it was location (*p* = .854, see Fig. S4). However, there were no significant differences between the No change and Change conditions for participants in the object-rotation condition, *ps* > .1. No other main effect or interaction was significant, either, *ps* > .1.


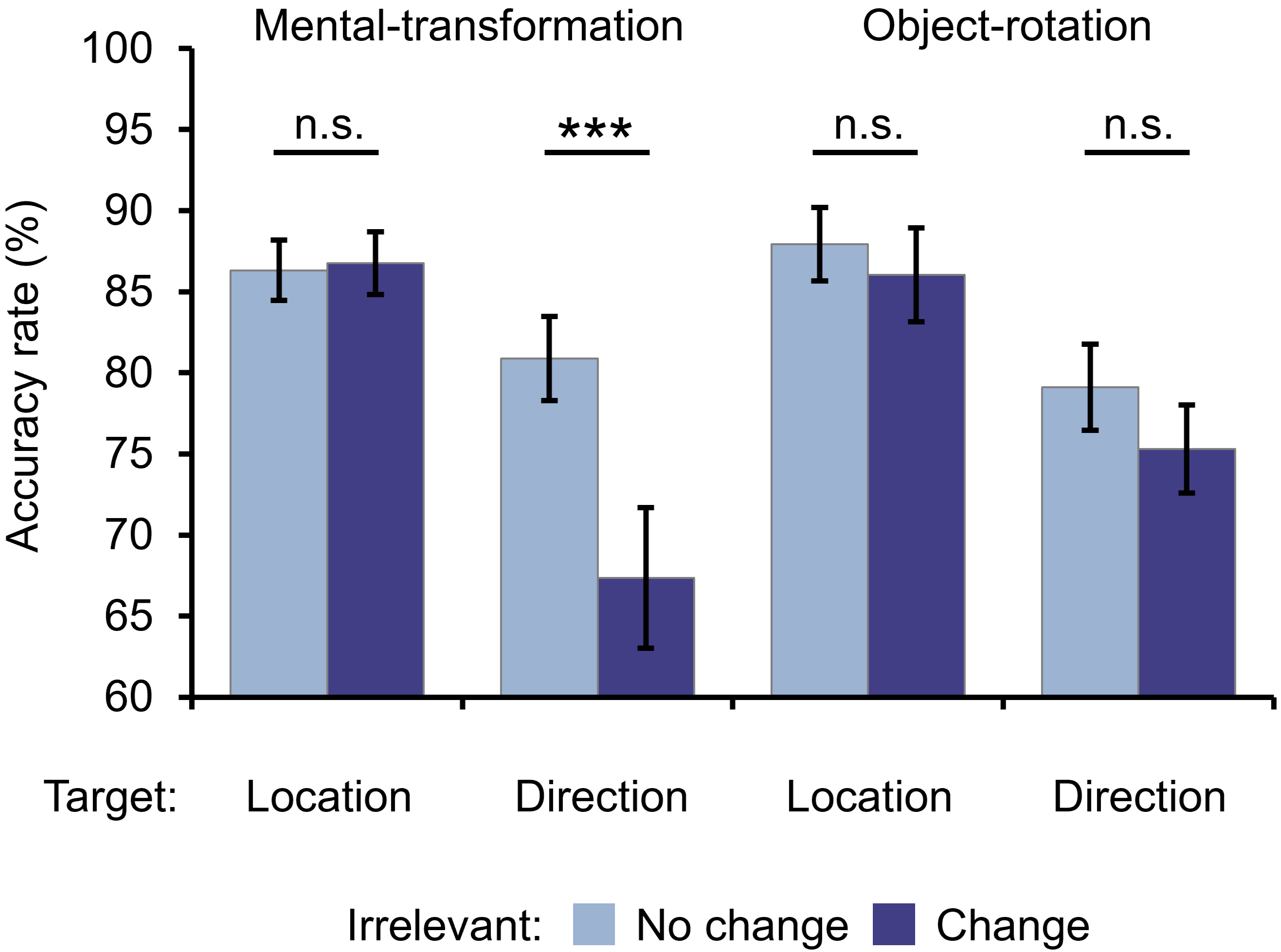


**Fig. S4** *Participants’ ACCs across different conditions in the target-present trials in Experiment 4.*

*Egocentric bias*

For the mental-transformation group, in 3.309% (*SD* = 3.341%) and 8.677% (*SD* = 4.923%) of the trials under the location and orientation condition, respectively, participants mistakenly identified target features that they had previously seen from their own perspectives in the memory array as appearing from the avatar’s perspective (referred to as Egocentric Error Rates). These errors accounted for 32.428% (*SD* = 23.322%) and 39.871% (*SD* = 15.928%) of the total errors in the two conditions (referred to as Egocentric Error Proportions). For the object-rotation group, in 3.088% (*SD* = 2.722%) and 8.456% (*SD* = 4.439%) of the trials under the location and orientation condition, respectively, participants mistakenly identified target features that they had previously seen from their own perspectives in the memory array as appearing from the avatar’s perspective. These errors accounted for 31.386% (*SD* = 20.181%) and 39.042% (*SD* = 16.274%) of the total errors in the two conditions.

We then conducted two mixed-measure ANOVAs on participants’ Egocentric Error Rates and Egocentric Error Proportions, respectively, both with Strategy (Mental-transformation vs. Object-rotation) as the between-participant variable and Target Dimension (Location vs. Orientation) as the within-participant variable. Both ANOVA analyses revealed significant main effects for Target Dimension. Participants showed both significantly higher Egocentric Error Rates (*F* (1, 66) = 103.689, *p* < .001, η_p_^2^ = .611) and significantly higher Egocentric Error Proportions (*F* (1, 66) = 6.353, *p* = .014, η_p_^2^ = .088) when recognizing the orientation as opposite to the location of the probe arrow. However, neither the main effects of Strategy nor the interaction was significant in either ANOVA analysis, *ps* > .5.
